# Construction of adenovirus vectors simultaneously expressing four multiplex, double-nicking guide RNAs of CRISPR/Cas9 and in vivo genome editing

**DOI:** 10.1038/s41598-021-83259-0

**Published:** 2021-02-17

**Authors:** Tomoko Nakanishi, Aya Maekawa, Mariko Suzuki, Hirotaka Tabata, Kumiko Sato, Mai Mori, Izumu Saito

**Affiliations:** 1 Laboratory of Virology, Institute of Microbial Chemistry (BIKAKEN), Microbial Chemistry Foundation, Shinagawa-ku, Tokyo, 141-0021 Japan; 2 Laboratory of Molecular Genetics, Institute of Medical Science, University of Tokyo, Minato-ku, Tokyo, 108-8639 Japan; 3 Department of Pharmaceutical Engineering, Faculty of Engineering, Toyama Prefectural University, Toyama, Japan; 4 Present Address: Department of Human Genetics, Institute of National Center for Child Health and Development, Setagaya-ku, Tokyo, 157-8535 Japan

**Keywords:** Hepatitis, Viral infection

## Abstract

Simultaneous expression of multiplex guide RNAs (gRNAs) is valuable for knockout of multiple genes and also for effective disruption of a gene by introducing multiple deletions. We developed a method of Tetraplex-guide Tandem for construction of cosmids containing four and eight multiplex gRNA-expressing units in one step utilizing lambda in vitro packaging. Using this method, we produced an adenovirus vector (AdV) containing four multiplex-gRNA units for two double-nicking sets. Unexpectedly, the AdV could stably be amplified to the scale sufficient for animal experiments with no detectable lack of the multiplex units. When the AdV containing gRNAs targeting the *H2-Aa* gene and an AdV expressing Cas9 nickase were mixed and doubly infected to mouse embryonic fibroblast cells, deletions were observed in more than 80% of the target gene even using double-nicking strategy. Indels were also detected in about 20% of the target gene at two sites in newborn mouse liver cells by intravenous injection. Interestingly, when one double-nicking site was disrupted, the other was simultaneously disrupted, implying that two genes in the same cell may simultaneously be disrupted in the AdV system. The AdVs expressing four multiplex gRNAs could offer simultaneous knockout of four genes or two genes by double-nicking cleavages with low off-target effect.

## Introduction

Genome editing using the CRISPR/Cas9 system is now widely used in various basic studies and in applied fields in vitro and in vivo^1–3^. The Cas9 enzyme recognizes both the protospacer adjacent motif (PAM) and its upstream 20-nucleotide (nt) target sequences specified by guide RNA (gRNA), and cleaves the target site.

Simultaneous expression of multiplex gRNAs is valuable for knockout of multiple genes. Also, double or multiple cleavages in one target gene are guaranteed to cause knockout of the target gene because of a large irreversible deletion^4,5^. In addition, a strategy called double nicking using the Cas9 nickase, a mutated Cas9 that introduces a nick instead of a cleavage, increases the cleavage specificity and reduce off target effects^6–8^. To cleave at the target site in this strategy, two nicks are introduced by Cas9 nickase using two gRNAs located preferably within 30 nts in the both strands. Because each gRNA specifies 20-nt sequences, 40-nt sequences in total, the cleavage specificity remarkably increases. However, two gRNAs are required for one cleavage in this approach. Therefore, if the simultaneous expression of four multiplex gRNAs is available, specific and efficient knockout of the target genes would become possible. Moreover, if adenovirus vectors, lentivirus vectors and AAV vectors expressing highly-multiplex gRNAs are available, they certainly contribute to more specific and efficient genome-editing therapy.

For multiplex gRNA expression, it is desirable that all of the gRNA-expressing units (gRNA units, hereafter) are connected in one molecule to achieve simultaneous expression in a single cell. However, direct repeat sequences in plasmids tend to be deleted even in *RecA*^*−*^ hosts of *E. coli*, owing to homologous recombination^9–11^. Also, all of the identical DNA fragments must be connected in the same orientation because a palindromic structure inhibits plasmid replication^12,13^. Using the Golden-Gate Assembly method^14^. Sakuma et al. produced a plasmid targeting seven different genes and simultaneously disrupted all of them. Also, Vad-Nielsen et al. constructed plasmids containing up to 30 gRNA expression units targeting ten genomic loci and achieved simultaneous inhibition of multiple endogenous genes reported^15,16^, Although this method is very convenient, the construction of 7 and 30 plasmids for each gRNA unit is needed. In this study, we took advantage of a lambda in vitro packaging system (lambda packaging, hereafter) for construction of multiplex-gRNA units, which has been used for the construction of genomic DNA libraries^17^. The transduction efficiency of plasmids containing a lambda cos sequence, called cosmids, is very high (about 5 × 10^9^ colonies/μg), and the procedure is as simple as that of transformation. In contrast to transformation, lambda packaging is size-selective and transfers only large cosmids typically between approximately 36 and 52 kilobases (kb) in size. Consequently, unwanted deleted cosmids generated by homologous recombination are selected out^18^.

Adenovirus vectors (AdVs) have several advantages as tools for genome editing. They can bear large DNA fragments up to 8 kb in contrast to AAV vectors and differ from lentivirus vectors in that the vector genome is not integrated into the cell chromosomes and is lost after editing. In addition, the mutation rate of the vector during amplification is about 100 times less than that of lentivirus vectors and RNA-based vectors^19,20^. These features appear to be preferable for the use of AdVs in genome-editing therapies. AdVs containing two multiplex-gRNA units have been reported^21^, although no reports have been published about the stability of more than two gRNA units inserted into AdVs. Another feature of AdVs is that, when they are intravenously administered to adult mice, they are very efficiently transduced to the liver, and most of the hepatocytes are infected and expresses the gene products^22^. Consequently, when mice are administered with two different AdVs as a mixture, a significant portion of the liver cells might doubly be infected, and the gene products encoded in both AdVs, such as Cas9 and gRNAs, could simultaneously be expressed in the same cell.

We here describe a method of “Tetraplex-guide Tandem” for the one-step construction of stable cosmids containing four multiplex-gRNA units. Using this method, we constructed AdVs containing four multiplex-gRNA units. Unexpectedly, the AdVs could be amplified in 293 cells to a level sufficient for in vivo experiments with no detectable lack of the multiplex units. Moreover, the AdV expressing four gRNAs in two double-nicking settings simultaneously disrupted both individual target regions or generated one-large (bridged) deletions in liver cells in vivo. Therefore, the methods described here may contribute to safer and more efficient use of multiplex-gRNA units for genome editing therapy.

## Results

### Construction of cosmids containing four and eight multiplex-gRNA units in one step cloning

**Figure 1:**
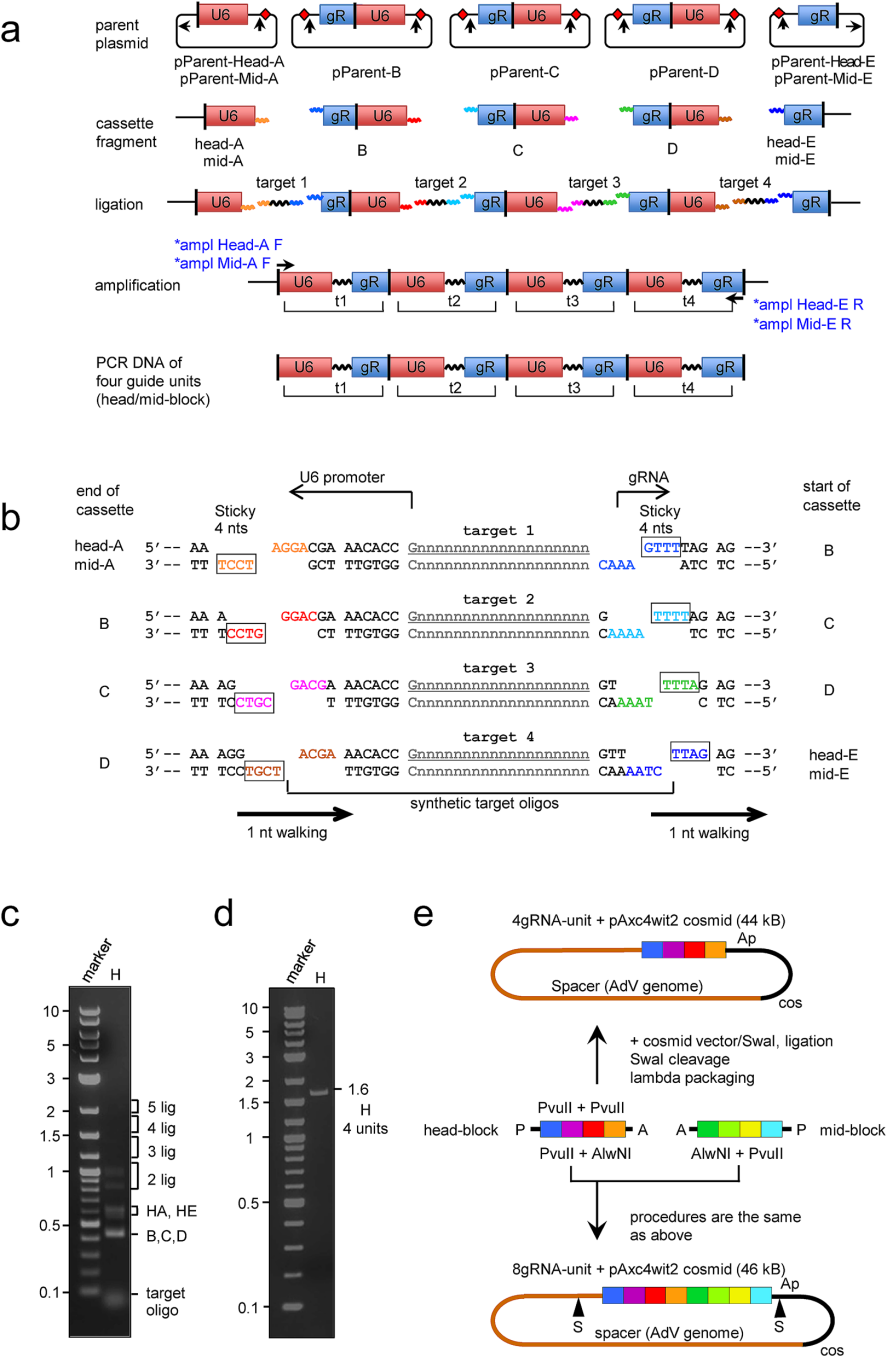
Schematic representation of the method for construction of cosmids containing four or eight multiplex-gRNA units in one step. (**a**) The process of construction of a block consisting of four multiplex gRNA-expressing units. This figure commonly shows for head- and mid-blocks, which can be connected. U6, U6 promoter; gR, gRNA scaffold. In the first row, eight red diamonds represent BsaI and the vertical arrows show cleavage positions to excise the cassette fragments. The horizontal arrows in the leftmost and rightmost plasmids indicate BglI, DraIII, and SapI sites used to obtain head/mid-A, head-E, and mid-E fragments, respectively. In the second and third rows, the specific sticky four nts produced by BsaI are shown as colored horizontal waves on both sides of the cassette fragments (the colors are identical to the boxed four nts in b, the detailed junction sequences are also shown in Supplementary Fig. S1a). The structures of the resultant cassette fragments of mid-A and mid-E are identical to those of head-A and head-E, respectively, as for BsaI-producing sticky ends, and hence the same cassette fragments of B, C, and D for head-block can be used for mid-block construction. In the fourth row, arrows show the amplifying primers to obtain the four-tandem gRNA units shown in the fifth row. The *ampl Head-A F-primer and *ampl Head-E R-primer containing AlwNI site were used to obtain head-block consists of four gRNA units containing head-t1 to -t4. For mid-block, *ampl Mid-A F-primer containing AlwNI site and *ampl Mid-E R-primer were used to obtain mid-block containing mid-t1 to -t4. The AlwNI sites included in *ampl Head-E R-primer and *ampl Mid-A F-primer, produce non-palindromic sticky ends, so that head-block and mid-block can be connected by orientation-specific ligation at the cloning step into a cosmid (see also e, lower). The primer sequences are shown in Supplementary Fig. S1a,b, and Table S1. Representations are described in the text. The reason of the names of head- and mid-blocks are that mid-block could be connected with tail-block using DraIII present in *ampl Mid-E R-primer in the future. We tried to obtain each cassette fragment by PCR using one of the pParent plasmids shown in a, first row. However, fragments of the head/mid blocks cloned in the cosmid frequently contained mutation, because PCR amplification twice doubled the PCR errors. (**b**) Structures of 3′ terminal of U6 promoter, the target oligonucleotide sequences, and 5′ terminal of gRNA scaffold. Boxed colored nucleotides are the sticky specific four nts at the end of each cassettes. Complementary sequences of the sticky four nts to them (unboxed) are present in both ends of the target oligos. The four nucleotides at both ends of the target oligos move stepwise by one nucleotide (target walking). The terminals of these oligos were designed to compensate for the lack of nucleotides of the U6 and gRNA-scaffold sequences, and hence their sequences are “repaired” to restore their intact form by ligation using their specific four-nt sticky ends. The 5′ nucleotides of the 20-nt targets are tentatively G in this figure, because 5′G is the most efficient in the expression of U6 promoter. (**c**) Preparation of the head block consisting of four multiplex-gRNA units. Lane H, ligation products of all five cassettes including head-A/head-E and four target oligos, respectively. “5 lig” to “2 lig” show the regions of connected cassette fragments plus target oligos. The numbers from “2 lig” to “5 lig” indicate those of connected cassettes; the target oligos are ignored because they are only 20 nt long. Bands in the regions of 4 lig and 5 lig are not seen in this gel. Full-length gels are presented in Supplementary Fig. S10. (**d**) An amplified fragment consisting of gRNA units containing t1-t4. Lane H, the 1.6-kb band shows the amplified head-block consisting of four units. Full-length gels are presented in Supplementary Fig. S10. (**e**) The process of construction of cosmids containing multiplex-gRNA units. For four gRNA units, head-block (center left, four squares from blue to orange) was cleaved with PvuII and was cloned into SwaI site of the pAxc4wit2 (top). For eight gRNA units, we newly prepared eight synthetic oligos for four gRNA units for mid-block (center right, four squares from green to light blue), in accordance with the same strategy of head-block construction but using mid-A and mid-E fragments. In the ligation step, the expected 2.2-kb band was also invisible as well as head-block, but we also obtained the 1.6-kb mid-block fragment after the PCR amplification (data not shown). Then, the amplified DNAs of both head and mid-block were doubly cleaved with AlwNI for specific ligation of both blocks and PvuII, which produced blunt ends suitable for cloning into SwaI site of the vector. After the ligation of both blocks with the SwaI-linearized pAxc4wit2, lambda packaging was performed to obtain the cosmid containing eight gRNA units (bottom). The detailed methods for cosmid construction are described in detail in the “Supplementary Materials and Methods”. P, PvuII; A, AlwNI; S, SspI. Although SspI cuts at several sites, only two sites flanking the eight-unit array in the cosmid are shown. The adenovirus genome of pAxc4wit2 lacks 2873 base pairs (bp) in E1 region (positions 455–3328) and 1877 bp in E3 region (28,593–30,470) and possesses an E4 SwaI insertion site at position 35,770.

We developed a method for construction of a DNA fragment consisting of four multiplex-gRNA units in one step (“Tetraplex-guide Tandem”) (Fig. 1a, bottom, called head-block) by using only PCR without plasmid cloning. Because the aim of this study is the construction of AdVs expressing multiplex-gRNA units, we introduced the fragment into a cassette cosmid pAxc4wit2^23^ for construction of replication-deficient adenovirus vector.

This method is unique in that, an expression unit consisting of U6 promoter, 20-nt target, and gRNA scaffold connected in this order is not constructed; instead, a non-functional unit of gRNA scaffold and U6 promoter connected in this order was used. First, we constructed five parent plasmids: pParent-Head-A, pParent-B, pParent-C, pParent-D and pParent-Head-E with specific sticky 4-nt sequences produced by BsaI at one or both ends (Fig. 1a, first row, eight red diamonds; sequences around the BsaI sites are shown in Supplementary Fig. S1a) to produce five cassette fragments, head-A, B, C, D and head-E (second row). The sticky 4 nt are shown as specifically-colored horizontal waves on the right side of cassette head-A, on the both sides of the cassette fragments B, C and D, and on the left side of cassette head-E (Fig. 1a, second row; the 4-nt sequences of the specifically-colored waves are shown in eight boxes in Fig. 1b).

Next, we prepared four synthetic fragments by annealing oligonucleotides (oligos), containing the target sequences of 20 nt (Fig. 1a, third row, target 1 to target 4; Fig. 1b, center, target 1 to target 4, gray G19nt of the top strand and C19nt of the bottom strand). These four target sequences are tagged with several nucleotides including 5′-terminal 4 nt, which are complementary to the sticky 4 nt of U6 promoter (for example, Fig. 1b, first row, left, orange 5′-AGGA for target 1, and sticky 4 nt of boxed TCCT-3′ at the end of cassette head-A, respectively). Similarly, these target sequences are tagged with the nucleotides at its 3′-terminal complementary to the sticky 4 nt of gRNA scaffold (for example, first row, right, blue CAAA-3′ for target 1, and sticky 4 nt of boxed 5′-GTTT as the 5′ end of cassette B, respectively). The both terminal sequences of these four target fragments sequentially differed by one nucleotide (“1 nt walking” in Fig. 1b, bottom).

Finally, the five cassettes and four annealed target oligos (Fig. 1a, third row) were ligated at once. Because the eight sticky ends consisted of four nucleotides differ from each other, specific ligation can simultaneously connect all of the nine DNA fragments of cassette head-A, target 1, cassette B, target 2, cassette C, target 3, cassette D, target 4 and cassette head-E (Fig. 1a, third row); consequently, the desired structure of the four expression units containing of target 1, target 2, target 3, and target 4 can be generated (Fig. 1a, fourth row; Fig. 1c). Although the expected band of 2.3 kb containing all four gRNA units was not visible after electrophoresis (Fig. 1c, 5 lig), the gel piece of this size was excised to extract DNA and PCR was performed using the forward primer of head-A cassette and the reverse primer of head-E cassette (Fig. 1a, fourth row, black arrows of *ampl Head-A F-primer and *ampl Head-E R-primer; their sequences are shown in Supplementary Table S1). We obtained the amplified 1.6-kb DNA (Fig. 1d) consisting of four desired multiplex-gRNA units (fifth row) lacking plasmid tags upstream of the head-A and downstream of head-E cassettes (fourth row, thin horizontal lines at left and right terminals). This result showed that even an undetectable amount of the ligated 2.3-kb DNA was sufficient to obtain the desired four multiplex-gRNA units.

The amplified product, called head-block (Fig. 1a, bottom), was cloned into SwaI site of the pAxc4wit2 cosmid vector to produce a cosmid containing the four gRNA units (Fig. 1e, upper). Thus, the cosmid containing four multiplex-gRNA units was successfully obtained in one step using an in vitro packaging system. The identical cosmid could also be constructed (Supplementary Fig. S2) by ligating two fragments, each containing two gRNA units (head t1 + t2 and head t3 + t4) into pAxc4wit2 vector.

Moreover, the head-block is designed so that it can be connected with another block (mid-block) containing four other multiplex-gRNA units, and that a cosmid bearing eight gRNA units can be obtained in a single cloning step (Fig. 1e, lower). To obtain mid-block we constructed two parent plasmids, pParent-Mid-A and pParent-Mid-E (Fig. 1a, first row, identical to the pParent-Head-A and pParent-Head-E in this figure), containing the sequences for amplifying primers *ampl Mid-A F and *ampl Mid-E R (Fig. 1a, fourth row, arrows under the *ampl Head-A F and *ampl Head-E R; their sequences are shown in Supplementary Fig. S1b). Using these primers, mid-block consisting of four multiplex-gRNA units containing new target sequences was produced (Fig. 1a, bottom, identical to head-block in this figure). To produce a cosmid containing eight gRNA units (Fig. 1e, lower), the amplified DNAs of both head- and mid-blocks were doubly cleaved with AlwNI and PvuII and cloned into SwaI site of the pAxc4wit2 cosmid vector in one step. Examination of the cosmid structure using restriction enzymes showed that no apparent band due to deletion caused by homologous recombination was observed (Supplementary Fig. S3), indicating that a cosmid containing eight multiplex-gRNA units was stable. Lack of mutations was verified by sequencing using primers shown in Supplementary Table 1 and Supplementary Fig. S1.

### Stability of adenovirus vectors containing four multiplex-gRNA units

**Figure 2:**
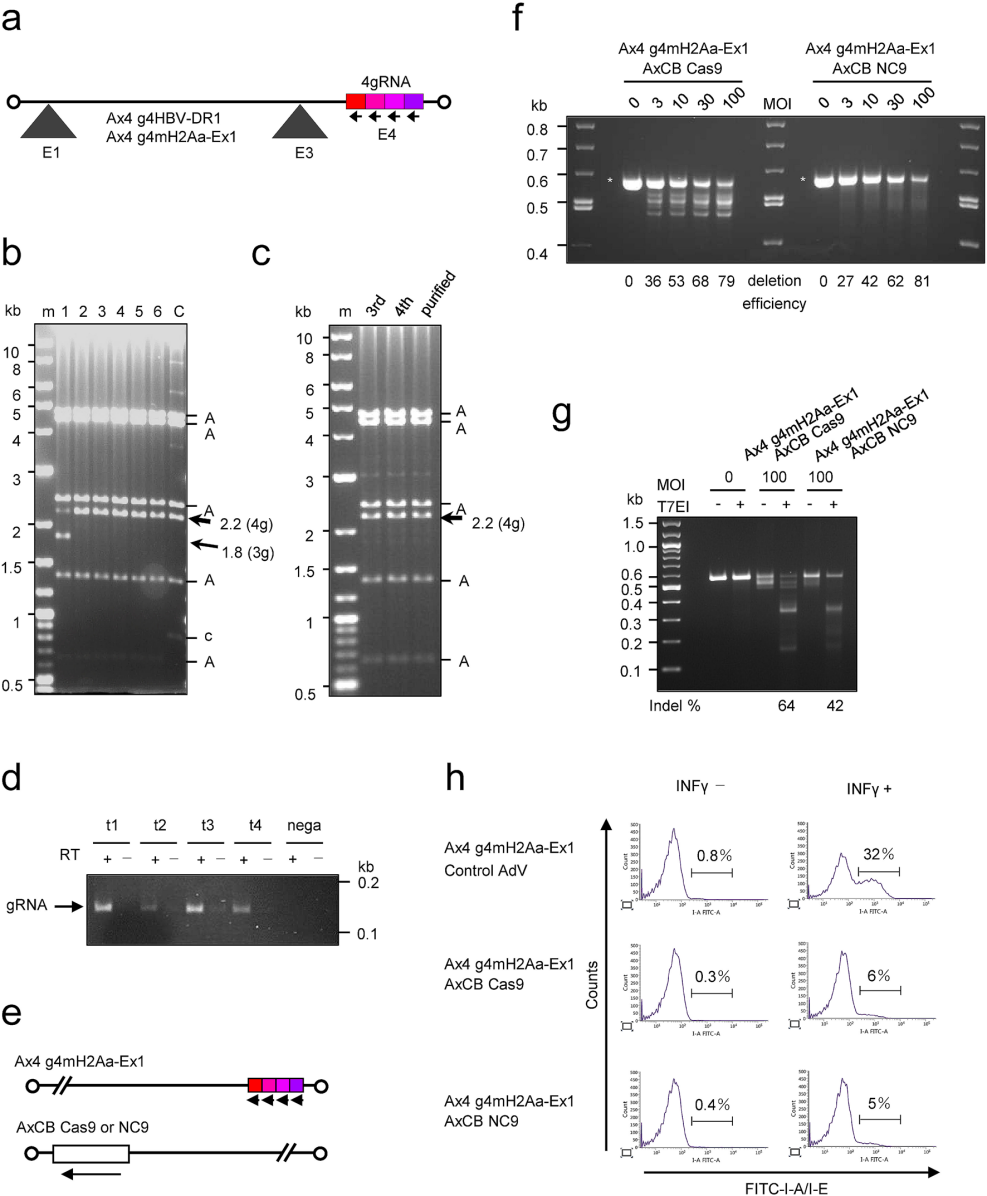
Production of adenoviruses (AdVs) containing four multiplex-gRNA units. (**a**) Structure of the AdVs. The four gRNA units are inserted at the E4 site in the leftward orientation. Four small arrows, direction of U6 promoters; black triangles, E1 and E3 deletion of the AdV genome; white circles, AdV terminal proteins. (**b**) The BspEI-cleaved AdV genome derived from 293 cells infected with the first stock. Lane m, marker; lanes 1 to 6, clone number; lane C, the parent cosmid containing the AdV genome. A, AdV fragment derived from virus clones and the parent cosmid; c, the fragment of cosmid-AdV junction in the cosmid. The AdV genome contains four multiplex units (4 g) of gRNAs targeting the DR1 region of the HBV genome. The total cellular DNA of 293 cells infected with the AdV of the first stock was digested with BspEI. Because the copy numbers of the AdV genome were very high, the vector DNA fragment can be seen without separation to the cellular DNA. Since the BspEI recognition sequence contains CG dinucleotide, which is uncommon in mammalian DNA, most of the cellular DNA fragments are very large and present as bands corresponding to > 10 kb in this gel. Full-length gels are presented in Supplementary Fig. S10. (**c**) The BspEI-cleaved AdV genome derived from infected cells with the third, fourth, and purified stock. The purified stock for in vivo experiments was prepared by infection of the fourth stock with 293 cells of a large-scale culture: therefore, the purified stock is not a concentrated fourth stock but an expanded fifth stock. Full-length gels are presented in Supplementary Fig. S10. (**d**) Expression of four multiplex gRNAs from the AdV containing four multiplex-gRNA units. Reverse Transcription-PCR was performed from total RNAs extracted from HepG2 cells infected with the Ax4g4mH2Aa-Ex1 (shown in a) at MOI 3. Full-length gels are presented in Supplementary Fig. S10. **(e)** Schematic representation of the AdV expressing four multiplex gRNAs targeting *H2-Aa* (Ax4g4mH2Aa-Ex1) and Cas9/Cas9 nickase (AxCB Cas9/NC9). Four small arrows and a large arrow show the direction of U6 and CB promoters. (**f**) In vitro genome editing using the AdVs shown in e. Mouse embryonic fibroblast (MEF) cells were infected with AdV expressing four multiplex gRNAs targeting *H2-Aa* and Cas9/Cas9 nickase at MOIs 3, 10, 30, and 100. The total cellular DNAs were isolated 3 days post-infection and were subjected to PCR amplification using primers HAAP28 and 29 (Supplementary Fig. S4a). Asterisks show intact 0.56-kb PCR fragments of the target *H2-Aa* region. Deletion efficiency is shown as decrease in the percentage of the band intensity of 0.56-kb fragments compared to MOI 0. Full-length gels are presented in Supplementary Fig. S10. (**g**) T7EI assay of MEF cells infected with the AdVs expressing four multiplex gRNAs targeting *H2-Aa* and Cas9/Cas9 nickase at MOI 100. Indel frequencies are shown below the lanes. Full-length gels are presented in Supplementary Fig. S10. (**h**) Flowcytometry analysis of MEF cells infected with the AdVs expressing four multiplex gRNAs targeting *H2-Aa* and Cas9/Cas9 nickase at MOI 30 followed by treatment with or without 300 U/ml INFγ. The cells were stained by FITC-conjugated anti-mouse I-A/I-E antibody. Representative histograms are shown by using FACSuite software.

We attempted to construct AdV containing four multiplex-gRNA units (Fig. 2a). Because, by chance, the size of the spacers for lambda packaging, between about 30 and 35 kb, is approximately the same as that of the AdV genome in the cassette cosmid pAxc4wit2^23^ used for the construction of AdVs. Therefore, the cassette cosmid can directly be used as a cassette for cloning multiplex-gRNA units. We first produced an AdV using the cosmid containing four multiplex-gRNA units targeting the DR1 region of the HBV genome, a region essential for DNA synthesis *in cis* (the target sequences of the four gRNAs are shown in Supplementary Table S2) into the E4 cloning site of the AdV cassette pAxc4wit2. After transfection, we isolated adenovirus clones, prepared the second stock, and examined the structure of the AdV genome by infecting 293 cells with aliquots of the vector stocks. Figure 2b shows a representative result; five clones out of six maintained four gRNA units intact and no band derived from deleted units was observed (lanes 2–6). Clone 1 (lane 1) produced a shorter band of 1.8 kb and its size exactly corresponded to three gRNA units, suggesting that one unit was deleted by homologous recombination during an early stage of vector replication in 293 cells.

We also constructed an AdV containing four multiplex-gRNA units targeting exon 1 of the mouse *H2-Aa* gene, a gene that encode the α-chains of the MHCII molecule I-A expressed in C57BL/6 mice (the target sequences of the four gRNAs are shown in Supplementary Table S2 and Fig. S4). The vectors of the second stock were sequentially amplified to obtain high titer stocks. Notably, the bands produced by deletion were hardly observed even in the fourth stock and its purified stock (Fig. 2c), showing that the four multiplex gRNA units on the AdV genome were maintained with no detectable lack. The titer of the fourth AdV stock was no less than that of normal AdVs, which is completely adequate to prepare purified viral stocks for animal experiments. The same result was obtained using the AdV containing four gRNA unites targeting HBV described above (data not shown).

### Genome editing in vitro utilizing AdV expressing four multiplex gRNAs

When gRNA expression from the AdV containing four multiplex gRNA units targeting *H2-Aa* region was examined, all four gRNAs were detected by Reverse Transcription-PCR in HepG2 cells two days after the AdV infection (Fig. 2d). We also produced AdVs expressing Cas9 nickase or native Cas9 under control of CB promoter, AxCBCas9 and AxCBNC9, respectively (Fig. 2e), and mouse embryonic fibroblast (MEF) cells were infected with the AdV expressing the four gRNAs together with the AdV expressing Cas9 or Cas9 nickase, followed by analyzing the target *H2-Aa* region by PCR. The AdVs were used for infection at multiplicity of infection (MOI) up to 100, because infection of AdV is generally inefficient in mouse cells.

In the PCR deletion assay, the intact 0.56-kb fragment obtained by using AdV expressing native Cas9 decreased and smaller fragments increased depending on the MOIs (Fig. 2f, AxCBCas9, lanes MOI 3 to 100). The deletion efficiency was 79% at MOI 100. The sizes of four major small fragments corresponded well to the length mediated by the deletions produced by four gRNAs (0.54, 0.52, 0.50 and 0.46 kb; explained in Supplementary Fig. S4), indicating that all four gRNAs were functional. In contrast, a smear was observed under the intact 0.56-kb fragment instead of distinct bands by using AdV expressing Cas9 nickase (AxCBNC9, lanes MOI 3 to 100). These results showed that the deletion sizes might not be uniform using Cas9 nickase compared to native Cas9 (see the last paragraph of the “Results” section) and also suggested that the smear may be characteristic in the PCR deletion assay of the multiplex double-nicking cleavages. The deletion efficiency by PCR assay was 81% at MOI 100 and is almost the same as that used native Cas9. The indels can be detected by T7EI assay, in which the PCR-amplified DNA fragment containing indels is denatured, annealed and treated with mismatch-specific T7 endonuclease I. The T7E1 assay was also performed, and the efficiencies using AxCBCas9 and AxCBNC9 at MOI 100 were 64% and 42%, respectively (Fig. 2g, lanes Cas9 T7E1+ and NC9 T7E1+). The higher efficiency of native Cas9 than Cas9 nickase using the T7E1 assay could be explained because native Cas9 would produce small indels generated by a single gRNA, while Cas9 nickase may hardly produce small indels since they may immediately be repaired (shown later in the sequencing data).

To examine the off-target mutation on the mouse genome, we selected the most possible off-target sequences of gRNAs targeting *H2-Aa* region in the mouse genome by CRISPRdirect^24^, and T7EI assay was performed using the total cellular DNA from the MEF cells infected with the AdV expressing the four gRNAs and Cas9 or Cas9 nickase at MOI 100 (Supplementary Fig. S5). Off-target mutagenesis was detected in one of the four off-target sequences using native Cas9, which was not detected using Cas9 nickase.

We also examined whether the *H2-Aa* gene expression was down regulated by infection of the AdV expressing the four gRNAs together with the AdV expressing Cas9 or Cas9 nickase. The *H2-Aa* gene encodes the α chain of the MHC class II molecule I-A and is presented on the cell surface by producing heterodimer with the β chain encoded by *H2-Ab* genes. Thus, *H2-Aa* gene expression level can be examined by staining the cells using the antibody against MHC class II molecule I-A. In MEF cells, the *H2-Aa* gene expression can be induced by INFγ^25^. When the MEF cells were treated with INFγ after infection of AdV expressing the four gRNAs together with control AdV at MOI 30, 32% of the cells became I-A positive (Fig. 2h). However, only 6 and 5% of the cells were I-A positive by AdV infection using AdV expressing Cas9 or Cas9 nickase instead of the control AdV. These results indicated that the *H2-Aa* gene was down regulated in approximately 80% of the cells because of the gene disruption.

Genome editing by AdVs expressing multiplex gRNA was also shown in HepG2 cells containing the chicken β-globin poly(A) region present in an expression unit of HBV X gene under the CAG promoter^26,27^. The cells were infected with an AdV containing four multiplex guide units targeting the poly(A) region together with the AdV expressing native Cas9 (Supplementary Fig. S6a, the sequences and positions of gRNAs are shown). The infection doses of AdVs were MOIs up to 10, because human cells are highly susceptible to AdV infection compared to mouse cells. The poly(A) region was examined by PCR (Supplementary Fig. S6b). The efficiency of irreversible deletion in PCR assay was 96% at MOI 10, though one gRNA (gRNA t3 in Supplementary Fig. S6a) appeared not to be functional. One reason of the high deletion efficiency is that the intact DNA can be disrupted when any combination of two gRNAs out of four are functional (illustrated in Supplementary Fig. S6c), suggesting an advantage of multiplex gRNA strategy. Thus, gene disruption by AdVs expressing multiplex gRNAs would be useful for efficient genome editing in vitro.

### Application of AdV expressing four multiplex gRNAs to in vivo genome editing

**Figure 3:**
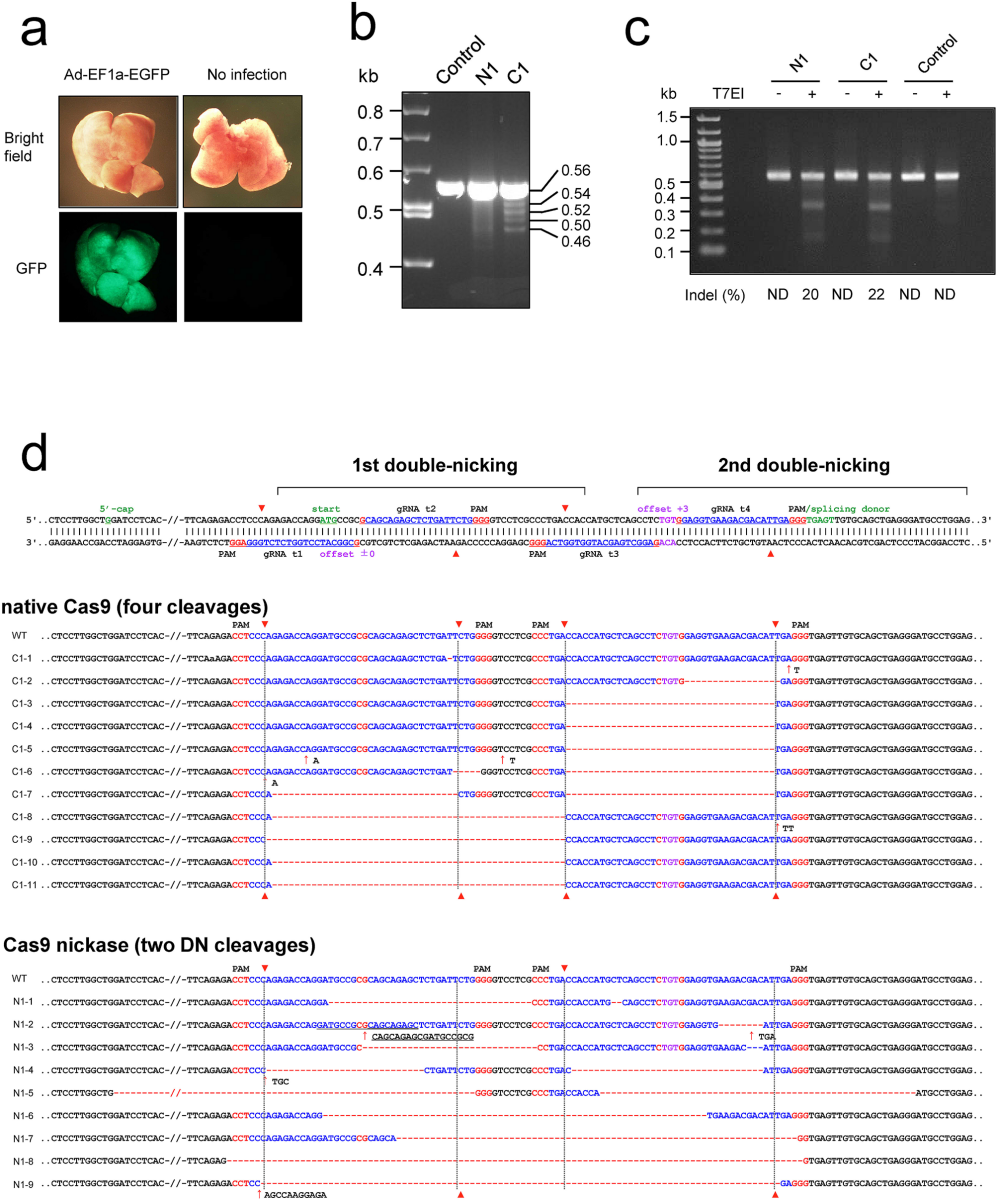
In vivo genome editing of mouse liver cells administered with AdV expressing four multiplex gRNAs targeting *H2-Aa*. (**a**) EGFP expression in the liver following the administration of AdV expressing EGFP under the control of EF1α promoter. Neonatal mice were administered at 0.5 × 10^8^ TCID_50r_ dose via the facial vein and dissected after 3 days. (**b**) PCR analysis of the liver genomic DNA from mice administered with AdV expressing four multiplex gRNAs targeting *H2-Aa*. AdV expressing four multiplex gRNAs targeting *H2-Aa* was administered (Ax4 g4mH2Aa-Ex1, 1.0 × 10^8^ TCID_50r_ dose) via the facial vein into neonatal mice with AdV expressing Cas9 nickase (AxCB NC9) or native Cas9 (AxCB Cas9) at 1.0 × 10^8^ TCID_50r_ dose. The liver genomic DNAs were isolated 2 weeks after administration and were subjected to PCR amplification using primers HAAP 28 and 29 (Supplementary Fig. S4a). C1, mouse administered with AdVs expressing four multiplex gRNAs and Cas9: N1, mouse administered with AdVs expressing four multiplex gRNAs and Cas9 nickase: Control, untreated C57BL/5 J mice. Full-length gels are presented in Supplementary Fig. S10. (**c**) Mutagenic efficiencies analyzed by T7EI assays in mouse liver cells. Genomic DNAs isolated from liver cell of mouse C1 and N1 were subjected to T7EI assays. The control consisted of DNA from untreated C57BL/5 J mice. Indel frequencies are shown below the lanes. ND, not detected. Full-length gels are presented in Supplementary Fig. S10. (**d**) The indel sequences in the two double-nicking target regions of the *H2-Aa* gene by administration of AdV expressing four multiplex gRNAs together with Cas9 nickase. (Upper row) The double-nicking target positions and sequences in exon 1 of the mouse *H2-Aa* gene. In the first double-nicking region, the gRNA target 1(t1) and PAM sequences of 5′-NGG (blue and red, respectively) in the bottom strand specifies the nick position (red triangle) in the top strand at 3 nt downstream of PAM. Also, the gRNA t2 and PAM in the top strand specified the nick position in the bottom strand. The offset (pink) is the length between the 5′-terminals of the pair of target sequences in the double-nicking region; in the first region the offset length is, by chance, 0 nt, and the two nicks produce 5′-overhangs of 34 nt in this case. Efficient cleavage occurs when the offset lengths are between − 5 and + 30^7^. Similarly, in the second double-nicking region the offset length is + 3 nt between the 5′-terminals of gRNA t3 and gRNA t4. The 5′-cap site, the start codon and the splicing donor site of exon 1 are shown in green. (Middle row) The indel sequences generated by native Cas9 in mouse C1, which produces double-strand cleavage with blunt ends. C1-1 to C1-11 are clones, which contain indels. The deleted regions are shown as red broken lines, and insertions are indicated as red upper arrows together with inserted nucleotides, such as ↑T. The deletions occurred almost precisely at the cleavage sites and, consequently, the two blunt-ends joined. (Lower row) The indel sequences generated by Cas9 nickase D10A in mouse N1. N1-1 to N1-9 are clones, which contain indels. Presentations are the same as in middle row. In the first and second double-nicking regions, 5′-overhangs of 34 and 40 nt, respectively, must be produced and, subsequently, cleavages occur. The annealed sequences restore the target sequences and are recleaved until the target sequences are destroyed.

To explore the genome editing efficiency of the AdV expressing all four multiplex gRNAs in vivo, we chose mouse neonates, because their body weight is much lower than that of adults, editing effects in liver could be achieved using only small amount of AdVs. It has been reported that a high transgene expression in the liver was observed in the neonatal mice, though the expression levels in the neonatal liver were approximately fivefold lower than those in the adult liver^28^. To confirm the feature of AdV that the liver cells can efficiently be transduced through the intravenous injection, we first administered the AdV expressing EGFP under the control of EF1α promoter^29^ into newborn mouse pups through the facial vein. The whole liver was stained by EGFP 3 days after infection with 1.0 × 10^8^ TCID_50r_ dose (Fig. 3a and Supplementary Fig. S7).

For in vivo genome editing, we chose mouse *H2-Aa* gene as an example for targeting the genome. This gene is one of the major histocompatibility complex and is not expressed in the liver; knockout of the gene would have no influence on liver functions, such as cell growth advantage, which may disturb evaluation of genome-editing efficiency. The AdV (1.0 × 10^8^ TCID_50r_ dose) expressing the four multiplex gRNAs targeting the *H2-Aa* gene was administered together with the AdV of the same dose expressing native Cas9 or Cas9 nickase into newborn mouse pups (mice C1 and N1, respectively) through the facial vein. When genome indels (insert/deletions) in the mouse liver were examined 2 weeks after infection by genomic PCR (Fig. 3b), four major small fragments (0.54, 0.52, 0.50 and 0.46 kb) were detected under the intact 0.56-kb fragment using native Cas9 (mouse C1) and a smear was observed under the 0.56-kb fragment using Cas9 nickase (mouse N1) as well as observed in mouse embryonic fibroblast (MEF) cells (Fig. 2f and Supplementary Fig. S4). The results of PCR analyses of another one (mouse C2) and three mice (mice N2 to N4) derived from native Cas9 and Cas9 nickase administration confirmed that native Cas9 and Cas9 nickase produced the discrete four bands and the smear, respectively, both in vitro and in vivo (Supplementary Fig. S8a).

By T7EI assays, the indel rates were found to be 22% (mouse C1) and 20% (mouse N1) for AdVs expressing native Cas9 and Cas9 nickase, respectively (Fig. 3c). The additional one and three mice (C2 and N2 to N4) derived from native Cas9 and Cas9 nickase administration were shown in Supplementary Fig. S8b. The indel rates were 17% for mouse C2 and 27, 35, and 16% for mice N2, N3, and N4, respectively. The results again showed that the knockout efficiencies of Cas9 nickase appeared to be comparable to that of native Cas9 using the same set of four gRNAs, namely, two double-nicking gRNA-pairs.

The PCR-amplified DNAs containing the target region were cloned and the indels were examined by sequencing (Fig. 3d). When 80 clones each were examined for mice C1 and N1, eleven and nine clones contained indels for native Cas9 and Cas9 nickase, respectively. These results showed that all four gRNA targets were functional for both native Cas9 and Cas9 nickase. Native Cas9, which produces double-stranded cleavage, generated indels of a few nt that were located at or immediately adjacent to the predicted Cas9 cleavage sites (Fig. 3d, middle: the insertions of 1 and 2 bp in clones C1-1, C1-6 and C1-8, and the deletions of 1, 5, and 17 bp in clones C1-1, C1-6 and C1-2, respectively). Moreover, as expected, the set of two cleavage sites produced precisely predicted deletions^30^ (Fig. 3d, middle: 33-bp deletion in clone C1-7, exact 37-bp deletions in five clones from C1-3 to C1-7 and 42- or 43-bp deletions in four clones from C1-8 to C1-11). The results of six clones from the additional mouse C2 were shown in Supplementary Fig. S9. In contrast, Cas9 nickase yielded unpredicted deletions of various lengths; that is, the junction positions were not necessarily the nicking sites, and some deletions were very large (Fig. 3d, bottom). Indels occurred in the both double-nicking sites in all nine clones, showing that the gene was disrupted at the both double-nicking sites. Similar results were obtained for twelve clones, in total, from another mice N2 to N4 (Supplementary Fig. S9).

## Discussion

Here, we showed that, using lambda in vitro packaging, the cosmids containing four and eight multiplex-gRNA units could be constructed in one step. Moreover, we showed that the AdVs containing four gRNA units were very stable and that the vector could be amplified up to the scale needed for in vivo experiments.

Simultaneous expression of four multiplex gRNAs (two sets of double nicking) offered a complete disruption of the target gene by producing large deletions, as shown in Fig. 3d. Also, both the double-nicking sites were disrupted in most cases. Generally, it is difficult to examine whether the two cleavages actually occurred simultaneously, unless two DNA cleavage sites were present very near and within the range of one sequencing. These results could suggest that two separate genes might simultaneously be disrupted using AdV expressing four gRNAs. Double nicking strategy would be also useful, because it could reduce off-target cleavage using Cas9 nickase. Decrease in off-target effects using double nicking strategy needs to be examined.

The disruption efficiencies between native Cas9 and Cas9 nickase were almost comparable by PCR deletion assay (Figs. 2f and 3c), in agreement with a recent report using transfection in vitro^31^. However, by T7EI assay, indel efficiency was higher using native Cas9 compared to Cas9 nickase. This could be explained because native Cas9 would produce indels of a few nucleotides generated by a single gRNA. In fact, in the sequencing data in Fig. 3d and in supplementary Fig. S9 altogether, 14 small indels out of 17 clones (0.82 indels per clone) were observed using native Cas9, while only 4 small indels out of 23 clones (0.17 indels per clone) were detected using Cas9 nickase. Thus, PCR deletion assay misses indels of a few nucleotides, which are detected in T7E1 assay, and the editing efficiency obtained using native Cas9 was different from that using Cas9 nickase but was higher than the latter.

Notably, the patterns of gene disruption comparing native Cas9 and Cas9 nickase clearly differed when using the same set of four multiplex-gRNA units both in vitro and in vivo (Figs. 2f and 3b), and the sizes of deletions using Cas9 nickase were sometimes much larger than those using two cleavages of native Cas9 (Fig. 3d and Supplementary Fig. S9). Using native Cas9, the number of the clones possessing deletions accurately between the two cleavage sites produced by double cleavages (Fig. 3d) were more than that of the clones possessing small indels generated by the single gRNA, as reported^29^. Using Cas9 nickase, both of the target regions were simultaneously disrupted in 18 out of 21 clones in total with only three exceptions (clones N2-1, N3-2 and N3-3 in the Supplementary Fig. S9), and large bridging deletions were observed in almost half of the clones.

The genome editing efficiency of about 20% without selection advantage in vivo may not be necessarily high, considering that whole liver was efficiently stained with GFP (Fig. 3a). One reason is that liver cells of neonatal mice grows very rapidly so that the introduced AdV DNAs would become quickly diluted. We used co-infection of two AdVs, and in a single cell the genome editing does not occur if either AdV is lost before Cas9/Cas9 nickase and gRNAs reaches the concentration sufficient to start genome editing. Another reason may be that the half-life of Cas9-gRNA complex is short, within 24 h^32^, while GFP is very stable. Interestingly, in most cases, when one double-nicking site was disrupted, the other was simultaneously disrupted, although the disruption efficiencies in liver cells were only 20%. If simultaneous disruption would occur in random, the efficiency of simultaneous disruption could be 4%. A possible reason is that, once a cell received both AdVs expressing multiplex gRNAs and Cas9/Cas9 nickase, they express sufficient amount of all four gRNAs to disrupt both the target sequences, even if their copy numbers are low. The efficiency of simultaneous disruption seems important to investigate a cooperative function of two genes. "True simultaneous disruption" of two genes might be a valuable character of our AdV system.

AdVs possess features that are beneficial for genome editing as stated in “Introduction”. We here added another advantage that AdVs containing four multiplex-gRNA units were stable and could be used for both in vitro and in vivo experiments. As shown in Fig. 2A, we inserted the array of four multiplex-gRNA units into the cloning site in the E4 region of the adenovirus genome^26^. The reason for this is that the U6 promoter is more active at the E4 site than at the commonly used E1 site and the leftward (inward) orientation is preferable^33^.

The high stability of AdVs is unexpected since homologous recombination occurs in mammalian cells. AdV is initially generated by transfection of DNA containing its full genome in 293 cells. We think that one possible reason may be the size selection. In the viral isolation step, the copy numbers of initially generated AdVs are small, and the infectious viral particles infect the adjacent cells and multiply by repeating this cycle. Because only AdV genomes larger than about 80% of the original size (36 kb) can be packaged in the virus particle, deleted genomes must be selected out during this cycle. Therefore, adenovirus possesses the mechanism of “in vivo packaging” similar to lambda in vitro packaging, which probably contributes to the stability of multiplex-gRNA units. The second potential reason for the high stability is that, because AdV titers are very high, very many copies of AdV genomes are introduced into a single cell and minimize the number of replication cycles. This may explain the high stability after the second viral stock.

It is unknown at present whether the stability of multiplex-gRNA units can be obtained using all AdV vector systems, because Adex vectors (Saito et al.^34,35^) used in this work are structurally different from widely used AdVs. The E1 deletion of Adex vectors starts at position 455 nt and contain entire Ad5 packaging domain consisting seven A-repeat sequences^36^, while that of AdVs, for example, developed by Bett et al*.*^37,38^ starts at position 342 and misses the functional three A-repeat sequences (A V, A IV and A VII). Thus, Adex vectors, which possess full-packaging domain, may multiply faster than other vectors, which might contribute to the high stability of multiplex-gRNA units. Also, the Adex vectors are unique to show remarkably low immunogenicity^39,40^; the expression of human growth hormone lasted for six months using immunocompetent C57BL/6 mice^40^.

Furthermore, we succeeded in construction of Adex vectors expressing Cas9 and Cas9 nickase under the control of very strong CB promoter, AxCBCas9 and AxCBNC9, respectively. The CB promoter consists of the cytomegalovirus enhancer and the chicken β-actin promoter. Though the expression units of CB-Cas9 and CB-Cas9 nickase are easily obtained from the original pX330 and pX335 (Addgene), these AdVs have not been reported, possibly because they might be difficult to obtain using other AdV systems. The AxCBCas9 and AxCBNC9 will be available from RIKEN BioResorce Bank (https://dna.brc.riken.jp/en/rvd/adenoen, Clone Search). Also, all of the seven parental cassette plasmids shown in Fig. 1a will be available from Addgene. Study of a functional system, cancer development for example, are often difficult because of the presence of family genes possessing similar or overlapping functions or several pass ways. We think that if "all-in-one" AdVs expressing multiplex gRNAs together with Cas9 or Cas9 nickase become available, they could offer specific and more efficient simultaneous knockout in vivo.

## Methods

### Construction of head-block containing four gRNA units

One hundred and fifty pmol of each target oligonucleotide (oligo) was treated with ten units of T4-polynucleotide kinase in a volume of 20 μL for 30 min, and then complementary pairs of the oligos were annealed. Ligation for head-block construction was performed by mixing 125 nL of each of the four annealed target oligos, 120 ng each of the five cassette fragments, and 400 units of T4 DNA ligase in a volume of 20 μL overnight. The ligated DNA was applied to one lane of a TAE gel of 15 cm in length and electrophoresed at 120 V for 2 h. The excised gel piece containing an invisible amount of the 2.3 kb DNA was subjected to DNA extraction using Wizard SV Gel and PCR Clean-up kit (Promega Corp., Madison, WI, USA) and PCR amplification of the extract was performed for 18 cycles using Tks Gflex DNA polymerase (Takara Bio, Inc., Shiga, Japan). The amplified head fragment was digested with PvuII, followed by gel electrophoresis to remove primer DNA. The cleavage with PvuII could be omitted, because the 5′ end of the PCR fragment produced by some of the PCR polymerases can directly be ligated.

### Cosmid preparation

The PvuII-cleaved head-block was inserted into a unique SwaI site present in the E4 cloning position of the cosmid pAxc4wit2^23^ to construct a cosmid containing four gRNA units. For the construction of AdVs expressing Cas9 and Cas9 nickase under the control of the CB promoter (AxCBCas9 and AxCBNC9), the expression units were excised from pX330 and pX335, respectively, using XbaI and NotI, blunt-ended using Klenow polymerase and inserted into the SwaI site present in the E1 cloning position of pAxcwit2^34^. The leftward (outward) orientation was chosen to avoid possible aberrant splicing^40^. The methods for constructing a cosmid containing multiplex-gRNA units and for handling the cosmids are described in detail in the “Supplementary Materials and Methods S1”.

### Cell culture, transfection, and AdV infection

Human 293 and HepG2 cell lines, derived from the human embryonic kidney and human hepatoblastoma, respectively, were obtained from RIKEN BioResource Research Center (RIKEN BRC; Japan) Cell Bank. The 293 cells were cultured in Dulbecco’s Modified Eagles Medium (DMEM) (Kohjin bio, Inc., Saitama, Japan) supplemented with 10% fetal calf serum (FCS). The 293 cells constitutively express adenoviral E1 genes and support the replication of E1-substituted AdVs. HepG2 cells were kept in high glucose DMEM supplemented with 10% FCS. Lipofectamine LTX & PLUS Reagent (Thermo Fisher Scientific, Inc., MA, USA) was used for plasmid transfection. The transfection was performed according to the manufacturer’s protocol. After infection with AdVs, the cells were maintained in DMEM supplemented with 5% FCS.

### Isolation of AdV clones and titration

To produce AdV containing multiplex-gRNA units or Cas9-nickase-expressing unit, 293 cells were transfected with the PacI- or BstBI-linearized AdV genome in pAxc4wit2 and on the next day transferred to a 96-well plate. The virus clones were obtained within 2 weeks (first viral stock of 150–200 μL). Cells in the 24-well plates were infected with one half of the first stock to obtain the second stock. The amount of stock solution used for further stock amplification was 4 or 5 folds compared to usual, while overinfection causing toxicity was carefully avoided. AdVs used for mouse experiments were purified by two-step CsCl gradient according to the method of Kanegae et al.^41^. Briefly, a viral stock solution was overlaid on the discontinuous CsCl solutions of 2.2 M and 4 M in 10 mM HEPES (pH 7.4) and centrifuged at 25,000 rpm for 2 h using Beckman SW28 roter (first step gradient). The solution of virus particles in the visible band between 2.2 M and 4 M CsCl was mixed with an equal volume of saturated CsCl solution. Step solutions of 2.2 M and 4 M CsCl were overlaid and centrifuged at 35,000 rpm for 3 h using SW41 rotor. The collected virus solution was dialyzed overnight in 10 mM HEPES (pH 7.4), 1 mM EDTA or in PBS(−). The process of virus titration using qPCR was performed as described previously^23,42^. The TCID50r in this work is the same as the relative viral titer (rVT) in these references based on the TCID_50_ titer of control virus AxEFGFP. The rVT titers are normally equivalent to TCID_50_ and PFU, but more accurately reflect the copy numbers introduced into the target cells because they are not influenced by the gene product that may be toxic to 293 cells^42^.

### Reverse transcription-PCR

Total cellular RNAs were extracted from HepG2 cells infected at a multiplicity of infection (MOI) 3 with Ax4g4mH2Aa-Ex1 and incubated for 2 days post infection. First-strand cDNA was synthesized from 1 μg of total RNAs by a SuperScript III reverse transcriptase (Thermo Fisher Scientific, Rockford IL, USA) using oligo dT_20_ as a primer. A portion of the synthesized cDNAs was subjected to PCR using sense primers mH2-Aa ex1ATG-1d0-b1F (5′-AGGACGAAACACCGCGGCATCCTGGTCTCTGGG-3′), mH2-Aa ex1ATG-1d0-t2F (5′-GG ACGA AACACCGCAGCAGAGCTCTGATTCTGG-3′), mH2-Aa ex1ATG-6d3-b3F (5′-G ACGA AACACCGAGGCTGAGCATGGTGGTCAGT-3′) and mH2-Aa ex1ATG-7d3-t4F (5′-ACGA AACACC GGAGGTGAAGACGACATTGAGTT-3′), and an antisense primer gRNAamp (5′-AAAAGCACCGACTCGGTGCC-3′) for detection of four gRNA expression. The PCR cycling conditions were as follows: 94 °C for 1 min, followed by 32 cycles at 98 °C for 10 s, 60 °C for 15 s and 68 °C for 30 s. PCR products were separated by polyacrylamide gel electrophoresis and stained with ethidium bromide.

### In vitro and in vivo AdV infection

The study was performed in accordance with the recommendations of the ARRIVE guidelines (http://www.nc3rs.org.uk/page.asp?id=1357). Animal experiments were approved by the Institutional Committee for Animal Experiments in the Institute of Microbial Chemistry (Tokyo, Japan) and performed in accordance with the relevant guidelines and regulations to minimize animal suffering. Mouse embryonic fibroblast (MEF) cells derived from C57BL/6 mice in a 6-cm cell culture dishes were infected at MOI 3, 10, 30 and 100 and incubated for 3 days postinfection. The same amount of AdV expressing multiplex gRNAs and Cas9 nickase were used for infection. For the administration of AdV, 50 μl (1 × 10^8^ TCID_50r_) of the vectors were injected into the facial vein (intravenous injection) of newborn C57BL/6 mice (CLEA Japan, Inc.) using a 29G needle, which were euthanized on the indicated days after administration. Total cellular DNAs were prepared and amplified by PCR with Tks Gflex DNA polymerase (Takara Bio) using a primer set HAAP28 (5′-TTGGATCCAAGTCTGCAGCTGGCAACTTTGACG-3′) and HAAP29 (5′-AAAGAATTCTACACTACAGGTACAAAGGCTTCAG-3′). The PCR cycling conditions were follows: 94 °C for 1 min, followed by 30 cycles at 98 °C for 10 s, 65 °C for 15 s and 68 °C for 30 s. The samples were analyzed on agarose gels by ImageJ. The amplified PCR fragments were also cloned into a pBluscript II SK and were sequenced.

### T7 endonuclease assay

For the T7EI assay, 200 ng of the amplified PCR products were reannealed to form a heteroduplex in 1 × NEB buffer 2 using the following program: denaturation at 95 °C for 5 min, reannealing from 95 to 85 °C at − 2 °C/s, holding at 85 °C for 1 min, cooling from 85 to 25 °C at − 0.1 °C/s and holding at 25 °C for 1 min, followed by cooling to 4 °C. The samples were then subjected to T7 endonuclease I (NEB) at 37 °C for 15 min and analyzed on agarose gels. The gels were imaged and quantified by ImageJ. The occurrence of indels was calculated using the following formula as described previously^43^: indel (%) = 100 × [1 − (1 − F_cut_)^1/2^]. F_cut_ is the fraction of the cleaved PCR product.

### Flowcytometry analysis

Mouse embryonic fibroblast (MEF) cells derived from C57BL/6 mice in a 24-well cell culture plate were infected with AdVs at MOI 30 and incubated for 2 days postinfection, and then treated with 300 U/mL INFγ for 2 days. The cells were stained using FITC-conjugated anti-mouse I-A/I-E antibody (clone M5/114.15.2, Biolegend, San Diego, CA, USA), followed by fixation with 1% paraformaldehyde/PBS, and analyzed on a BD FACSLyric (BD Biosciences, San Diego, CA, USA). Data analysis was performed using FACSuite software (BD Biosciences).

## Supplementary Information

Supplementary Information.
