# Supplementary material for: Construction of adenovirus vectors simultaneously expressing four multiplex, double-nicking guide RNAs of CRISPR/Cas9 and in vivo genome editing
Source: Sci Rep. 2021 Feb 17;11:3961. doi: 10.1038/s41598-021-83259-0 (PMC7889857; doi:10.1038/s41598-021-83259-0)
Supplement: Supplementary file 1 — Supplementary Information. [file 41598_2021_83259_MOESM1_ESM.pdf]

SUPPLEMENTARY INFORMATION

Construction of adenovirus vectors simultaneously expressing four multiplex, double-nicking guide RNAs of CRISPR/Cas9 and *in vivo* genome editing

Tomoko Nakanishi, Aya Maekawa, Mariko Suzuki, Hirotaka Tabata, Kumiko Sato, Mai Mori, Izumu Saito

Supplementary Materials and Methods

Construction of the cosmid containing four and eight gRNA units

For construction of the cosmid containing four gRNA units, 2 μg of SwaI-linearized pAxc4wit2 was ligated at 15°C overnight with 300 ng of head-block in a volume typically of 15 μL. For eight gRNA units, 300 ng each of head- and mid-blocks were used for ligation. Very high concentration of these fragments, a minimum volume, and also high purity were important. Half of the ligated DNA was digested with SwaI in a volume of 70 μL to remove the colonies containing the parent pAxc4wit2 cosmids lacking the insert and only 1 μL was used for packaging. The lambda packaging kits used were Lambda Inn (Nippon Gene, Japan) or Gigapack Plus (Stratagene). A scale of one-fifth using the former kit was usually sufficient, thereby reducing the cost, which is comparable with transformation. The orientation of the array of eight gRNA units was examined by digestion of the cosmid with EcoRV. The entire sequences were determined using 12 primers listed in Supplementary Table S1.

To prepare a 50-mL scale culture, 1 μL of the miniprep cosmid DNA was directly used for lambda packaging: though miniprep cosmid DNA is circular, twentieth of the packaging DNA usually yielded about 10^3 colonies. Culture flasks were vigorously shaken to achieve good aeration as usually applied for preparation. Cosmid DNA was prepared using an alkali-lysis procedure.[1] In the step of phenol-chloroform extraction the DNA solution was shaken vigorously to remove thoroughly contaminated DNase, which in our experience is more important than taking care of mechanical breaking of the cosmids. The drying-up step of plasmid preparation was avoided. Therefore, after centrifugation of the ethanol precipitation, the remaining 70% ethanol on the cosmid pellet was removed with a piece of Kimwipe, and TE was immediately added. Handling of the cosmids was essentially the same as that of the plasmids, except that described above. Throughout the cosmid experiments whole colony was suspended in miniculture for miniprep to minimize the duplication times of the *E. coli* DH5α. In addition, scissor-cut tips of micropipettes except miniprep DNAs for checking of their structures were used throughout the handling of cosmid DNAs to avoid mechanical breaking of cosmid DNA, especially for concentrated or ligated cosmids. Cosmids were always stored at –20°C.

A very high concentration was critical for lambda packaging because tetramolecular ligation of the insert flanked with two cos-containing vectors is needed, while ligation for typical plasmid cloning involves a dimolecular reaction between the insert and the vector plasmid DNA. Tetramolecular ligation for construction of cosmid containing eight gRNA

units can be performed efficiently because the packaging efficiency is extremely high unless a very high concentration is achieved. SwaI recognizes 8 nt of ATTT/AAAT and produces blunt ends. Practically, any DNA fragment, unless it contains a SwaI site, can be cloned to the SwaI site, after the treatment of Klenow enzyme, ligation and the subsequent recleavage with SwaI to remove the clones lacking the insert. Therefore, the SwaI site might be more convenient than the polylinker consisting of multiple enzyme sites.

References

1. Sambrook JF, Russell DW. Molecular Cloning: A Laboratory Manual. Cold Spring Harbor, NY: Cold Spring Harbor Laboratory Press. 2001.

Supplementary Table S1

List of primers for sequencing of the gRNA units

position	name	sequence
cassette head-A amplify	*ampl Head-A F (30mer)	5′-gcg CAGCTGTCAAC GGCGTCAATTGCATGC-3′<br>PvuII/HincII BsaHI/MfeI/SphI
cassette B seq primer	*seq-B F* (29mer)	5′-AATT A CCA CCTGC TGG T CCATGG TGCGCA -3′<br>PflMI/BfuAI NcoI FspI
	seq-B R (29mer)	5′-AATT TGCGCA CCATGG A CCA GCAGG TGG T -3′<br>FspI NcoI PflMI/BfuAI
cassette C seq primer	*seq-C F* (26mer)	5′-AATT GAA GCTC TTC ATGCAT AGTACT-3′<br>XmnI/SapI NsiI ScaI
	seq-C R (26mer)	5′-AATT AGTACT ATGCAT GAA GAGC TTC-3′<br>ScaI NsiI XmnI/SapI
cassette D seq primer	*seq-D F* (28mer)	5′-AATT A GCA ACTGG TGC AGATCT GATATC-3′<br>BstAPI/BsrI BglII EcoRV
	seq-D R (28mer)	5′-AATT GATATC AGATCT GCA CCAGT TGC T-3′<br>EcoRV BglII BstAPI/BsrI
cassette head-E amplify	*ampl Head-E R (35mer)	5′-cgc CAGCTGTTGACGCCAGCAACTGTACA GGATCC-3′<br>PvuII/HincII/BsaHI/AlwNI/BsrGI BamHI
cassette mid-A amplify	*ampl Mid-A F (34mer)	5′-gcg CAGCTGTCAAC GGCGTCAGTTGCTG GCTAGC-3′<br>PvuII/HincII BsaHI/ALwNI NheI
cassette mid-E amplify	*ampl Mid-E R (31mer)	5′-cgc CAGCTGTTGACGCCACACGGTG GGATCT-3′<br>PvuII/HincII/BsaHI/DraIII BstYI
8g head-AlwnI-Mid junction	H-AlwNI-M-F (25mer)	5′- GGATCCTGTACAGTTGCTGGCTAGC -3′<br>BamHI/BsrGI AlwNI NheI
	H-AlwNI-M-R (25mer)	5′- GCTAGCCAGCAACTGTACAGGATCC -3′<br>NheI AlwNI BsrGI/BamHI

All the terminal sequences of the head and mid-blocks are PvuII/HincII/BsaHI because of the usage of primers *ampl Head-A F, *ampl Head-E R, *ampl Mid-A F, and *ampl Mid-E R for construction. PvuII and HincII produce a blunt end and can be cloned to SwaI site at the E4 cloning position of pAxc4wit2, while BsaHI produces 5′-CG overhang, which can be ligated with ClaI site at the E1 cloning position of pAxc4wit2. AlwNI connects Head and Mid blocks. The gRNA/U6 junctions of cassettes B, C, and D are polylinker sequences that are not homologous to either U6 promoter or gRNA scaffold. Therefore, they can be used as unique sequencing primers (seq-B F/R, seq-C F/R, and seq-D F/R) and also allow easy subcloning of desired single or double units. When eight gRNA units are included in the cosmid, it cannot directly be sequenced because primers in the cassette B, C and D are present twice. Thus, before sequencing, the head and mid-block were separately amplified using the forward and reverse primers containing AlwNI and AdV primers outside the array of eight gRNA units.

Supplementary Table S2

1) List of gRNA targets to DRI and DR2 regions of hepatitis B virus (HBV), genotype C

DR1 region

U1	Hbc80b-F	5′-AGGA CGA AACACC GCAGAGGTGAAAAAGTTGCA-3′
U1	HBc80b-R	5′-AAAC TGCAACTTTTTCACCTCTGC GGTGTT TCG-3′
U2	HBc81b-F	5′-GGAC GA AACACC GACATGAACATGAGATGATT G-3′
U2	HBc81b-R	5′-AAAA C AATCATCTCATGTTCATGTC GGTGTT TC-3′
U3	HBc82t-F	5′-GACG A AACACC GCTGTGCCTTGGGTGGCTTT GT-3′
U3	HBc82t-R	5′-TAAA AC AAAGCCACCCAAGGCACAGC GGTGTT T-3′
U4	HBc83b-F	5′-ACGA AACACC GAAGCTCCAAATTCTTTATA GTT-3′
U4	HBc83b-R	5′-CTAA AAC TATAAAGAATTTGGAGCTTC GGTGTT-3′

DR2 region

U1	HBc69b-F	5′-AGGA CGA AACACC GAAGCGAAGTGCACACGGTC-3′
U1	HBc69b-R	5′-AAAC GACCGTGTGCACTTCGCTTC GGTGTT TCG-3′
U2	HBc70b-F	5′-GGAC GA AACACC GAGGTGAAGCGAAGTGCACA G-3′
U2	HBc70b-R	5′-AAAA C TGTGCACTTCGCTTCACCTC GGTGTT TC-3′
U3	HBc71b-F	5′-GACG A AACACC GGTTTCCATGCGACGTGCAG GT-3′
U3	HBc71b-R	5′-TAAA AC CTGCACGTCGCATGGAAACC GGTGTT T-3′
U4	HBc72b-F	5′-ACGA AACACC GACCTGGTGGGCGTTCACGG GTT-3′
U4	HBc72b-R	5′-CTAA AAC CCGTGAACGCCCACCAGGTC GGTGTT-3′
U5	HBc73t-F	5′-AGGA CGA AACACC GTCTTATATAAGAGGACTCT-3′
U5	HBc73t-R	5′-AAAC AGAGTCCTCTTATATAAGAC GGTGTT TCG-3′
U6	HBc74b-F	5′-GGAC GA AACACC GACCTTGGGCAAGACCTGGT G-3′
U6	HBc74b-R	5′-AAAAC ACCAGGTCTTGCCCAAGGTC GGTGTT TC-3′
U7	HBc75b-F	5′-GACG A AACACC GTCCTCTTATATAAGACCTT GT-3′
U7	HBc75b-R	5′-TAAAAC AAGGTCTTATATAAGAGGAC GGTGTT T-3′
U8	HBc76t-F	5′-ACGA AACACC GATGTCAACGACCGACCTTG GTT-3′
U8	HBc76t-R	5′-CTAAAAC CAAGGTCGGTCGTTGACATC GGTGTT-3′

2) List of gRNA target sequences to mouse *H2-Aa* gene, exon 1.

u1	mH2-Aa ex1ATG-1d0-b1F	5′-AGG ACGA AACACC GCGGCATCCTGGTCTCTGGG-3′
u1	mH2-Aa ex1ATG-1d0-b1R	5′-AAAC CCCAGAGACCAGGATGCCGC GGTGTT TCG-3′
u2	mH2-Aa ex1ATG-1d0-t2F	5′-GG ACGA AACACC GCAGCAGAGCTCTGATTCTG G-3′
u2	mH2-Aa ex1ATG-1d0-t2R	5′-A AAAC CAGAATCAGAGCTCTGCTGC GGTGTT TC-3′
u3	mH2-Aa ex1ATG-6d3-b3F	5′-G ACGA AACACC GAGGCTGAGCATGGTGGTCA GT-3′
u3	mH2-Aa ex1ATG-6d3-b3R	5′-TA AAAC TGACCACCATGCTCAGCCTC GGTGTT T-3′
u4	mH2-Aa ex1ATG-7d3-t4F	5′-ACGA AACACC GGAGGTGAAGACGACATTGA GTT-3′
u4	mH2-Aa ex1ATG-7d3-t4R	5′-CTA AAAC TCAATGTCGTCTTCACCTCC GGTGTT-3′

The Unit positions, the name of oligos and their sequences are shown. In these oligonucleotides spaces are introduced according to Figure 2B, though the direction is from 5' to 3' in the reverse strand. U1 means unit 1, etc.; HBc, HBV genotype C; t and b, top and bottom strands; F and R, forward and reverse orientation based on that of U6 promoter.

Supplementary Figure S1

a

pParent-Head-A (=pbs-Head-A), cassette fragment head-A (BglI-BsaI 521bp)

```
    BglI       pBS           <-|*ampl Head-A F-primer ------->       U6        |        BsaI    |-> pBS
--gccattc/aggc--(236bp)--ctgcag ccc gcgCAGCTGTCAACGGCGTCAATTGCATG CAAGGT------AA /AGGA CGAGACCcgc ggg ggatcc--
         ↑                 PstI      PvuII-HincII-BsaHI-MfeI-SphI                ↑                          BamHI
```

pParent-B (=pbs-BNcoI), cassette fragment B (BsaI-BsaI 370bp)

```
   pBS    <-|    BsaI    |       gR        |<------ seq-B F/R-primer ------->|       U6       |         BsaI    |-> pBS
-- ctgcag ccc gcgGGTCTCG/ GTTTTAG AG--TTTTG AATTACCACCTGCTGGTCCATGGTGCGCA/AATT CAAGGT------AA A /GGAC GGAGACCcgc ggg ggatcc--
     PstI                ↑                        PflMI-BfuAI-NcoI-FspI                          ↑                         BamHI
```

pParent-C (=pge-CNsiI), cassette fragment C (BsaI-BsaI 367bp)

```
pGA       <-|    BsaI    |       gR       |<----- seq-C F/R-primer ----->|       U6        |        BsaI    |-> pGA
--gaattc gatt gcgGGTCTCG/ TTTTAG AG--TTTTG AATTGAAGCTCTTCATGCATAGTACT/AATT CAAGGT------AA AG /GACG AGAGACCcgc aatc actagt--
  EcoRI                  ↑                   XmnI-SapI-NsiI-ScaI                            ↑                       SpeI
```

pParent-D (=pge-DBglII), cassette fragment D (BsaI-BsaI 369bp)

```
pGA       <-|    BsaI    |       gR      |<----- seq-D F/R-primer ------>|        U6        |        BsaI    |-> pGA
--gaattc gatt gcgGGTCTCT/ TTTAG AG--TTTTG AATTAGCACTGGTGCAGATCTGATATC/AATT CAAGGT------AA AGG /ACGA GGAGACCcgc aatc actagt--
  EcoRI                  ↑                  BstAPI-BsrI-BglII-EcoRV                             ↑                        SpeI
```

pParent-Head-E (=pge-Head-E), cassette fragment head-E (BsaI-DraIII 585bp)

```
pGA       <-|    BsaI    |      gR      |BamHI-BsrGI-AlwNI-BsaHI-HincII-PvuII|->            pGA       DraIII
--actagt gatt gcgGGTCTCT/ TTAG AG--TTTTG AGGATCCTGTACAGTTGCTGGCGTCAACAGCTGgcg aatc gaattc--(470bp)--cacgta/gtg--
  SpeI                   ↑                 <------------- *ampl Head-E R-primer        EcoRI                 ↑
```

b

pParent-Mid-A (=pbs-Mid-A), cassette fragment mid-A (BglI-BsaI 525bp)

```
    BglI       pBS           <-|*ampl Mid-A F-primer ---------->|      U6        |        BsaI    |-> pBS
--gccattc/aggc--(236bp)--ctgcag ccc gcgCAGCTGTCAACGGCGTCAGTTGCTGGCTAG CAAGGT------AA /AGGA CGAGACCcgc ggg ggatcc--
                         PstI       PvuII-HincII-BsaHI-ALwNI-NheI                   ↑                          BamHI
```

pParent-Mid-E (=pbs-Mid-E), cassette fragment mid-E (BsaI-SapI 435bp)

```
pBS       <-|    BsaI    |      gR      | BstYI-DraIII-BsaHI-HincII-PvuII  |->          pBS        SapI
--ctgcag ccc gcgGGTCTCT/ TTAG AG--TTTTG AAGATCCCACCGTGTGGCGTCAACAGCTGgcg ggg ggatcc--(318bp)--gctcttcc/gct--
  PstI                  ↑                <----------- *ampl Mid-E R-primer      BamHI                 ↑
```

Supplementary Figure S1. Detailed structures of parent plasmids and primers. **(a)** Structures of pParent-Head-A, pParent-B, pParent-C, pParent-D and pParent-Head-E. (b) Structures of pParent-Mid-A and pParent-Mid-E

Supplementary Figure S2

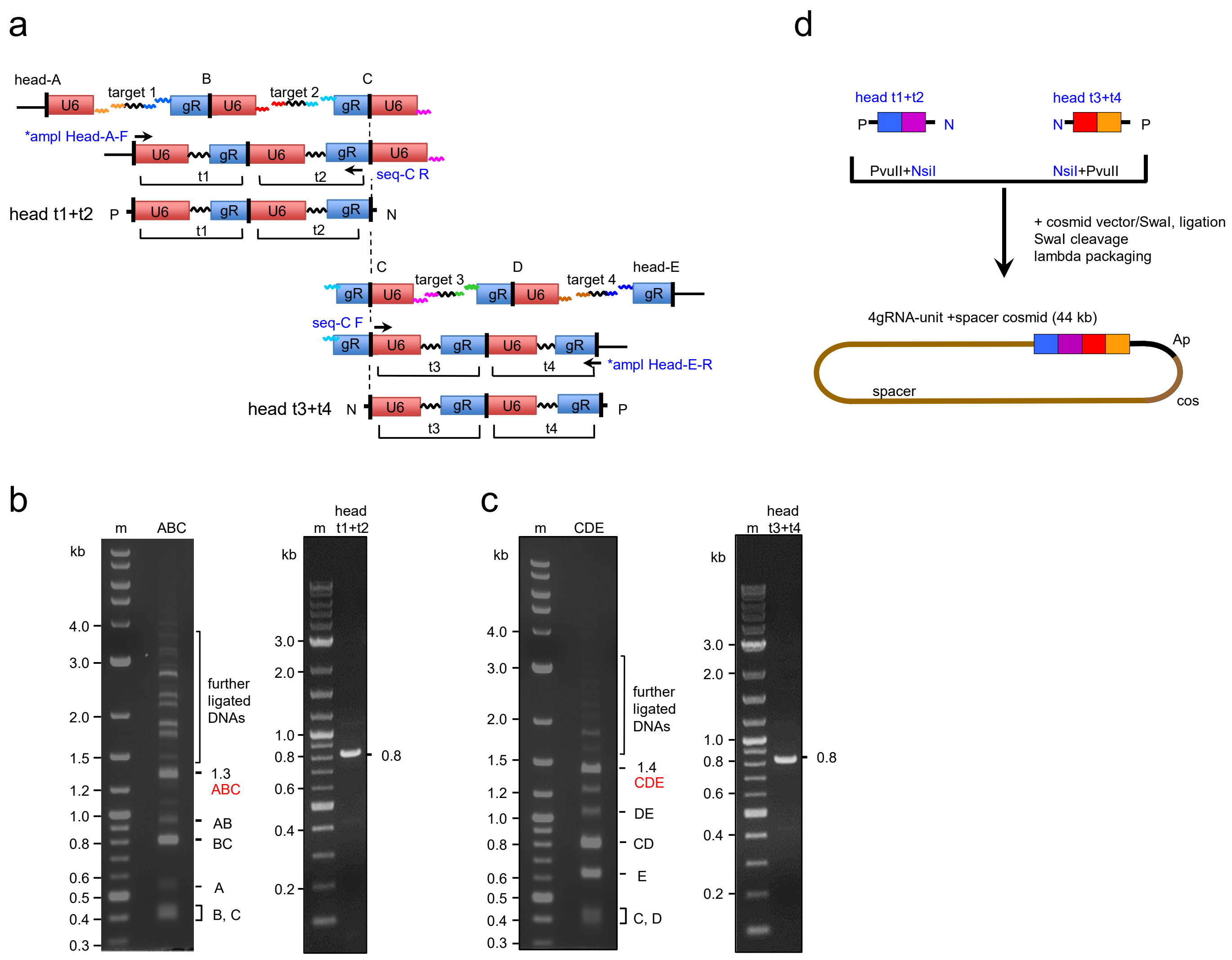

Supplementary Figure S2. A convenient construction of cosmid containing four gRNA units. **(a)** The strategy for the construction of the cosmids. Head t1+t2 fragment (third row) was constructed by five fragment ligation (first row) and amplification using the amplifying forward primer of head-A cassette and the reverse sequencing primer of cassette C containing an NsiI site (second row, *ampl Head-A F and seq-C R-primers, respectively). Similarly, the head t3+t4 fragment (sixth row) was constructed using the forward sequencing primer of cassette C containing the NsiI site and the reverse primer of head-E cassette (fifth row, seq-C F and *ampl Head-E R-primers, respectively). Sequences of the primers are shown in Supplementary Table S1. **(b)(c)** Ligation patterns of the three cassettes and two targets. Production of fragments containing two gRNAs is easier than that constructing a four gRNA-containing fragment as described in Fig. 1c and 1d. The desired ligation products containing cassette fragments of head-A, B, and C with targets 1 and 2 and cassette fragments of C, D, and head-E with targets 3 and 4 were abundant and easily visible in the ligation step. (b, left) Ligation pattern of head-A, B, C, t1, and t2. In this figure, t1 and t2 are ignored; for example, B means sole B, t1-B, B-t2, and t1-B-t2. The desired fragment of 1.3 kb was observed as a very dense band. The bands above 1.3 kb were generated because head-A fragment was obtained by the digestion of BsaI and EcoRV, which produces blunt ends. Therefore, head-A can be ligated with another head-A in the reverse orientation, forming CBA-ABC, for example. (b, right) Amplified fragment consisting of two gRNA units containing t1 and t2. (c, left and right) Results of similar experiments using C, D, head-E, t3 and t4. Full-length gels are presented in Supplementary Figure S10. **(d)** The process in construction of cosmid containing four gRNA units by ligating two fragments, each containing two gRNA units (head t1+t2 and head t3+t4). N, NsiI.

Supplementary Figure S3

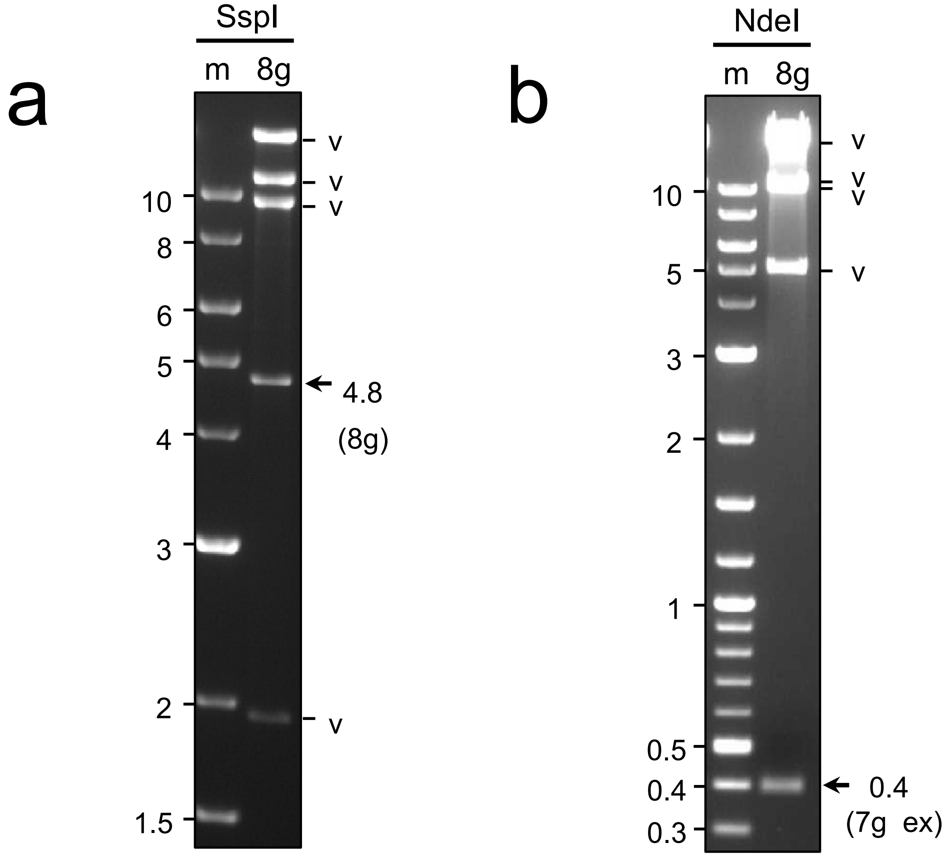

Supplementary Figure S3. Examination of the structure of the cosmid containing eight gRNA units. **(a)** The SspI-cleaved cosmid containing eight gRNA units. SspI cuts several sites in the cosmid in addition to two sites flanking the eight-unit array shown in Fig. 1E. After SspI cleavage, a 4.8-kb band corresponding to the fragment of eight intact multiplex units (8g) was observed. No apparent band due to deletion caused by homologous recombination was observed below this band. v, vector-derived DNA fragment. Full-length gels are presented in Supplementary Figure S10. **(b)** The NdeI-cleaved cosmid containing eight gRNA units. Because NdeI cleaves the gRNA unit at a single site, seven unit-length fragments of 0.4 kb were excised (7g ex) from the array of the eight tandem units. No other bands due to rearrangements were detected. Full-length gels are presented in Supplementary Figure S10.

Supplementary Figure S4

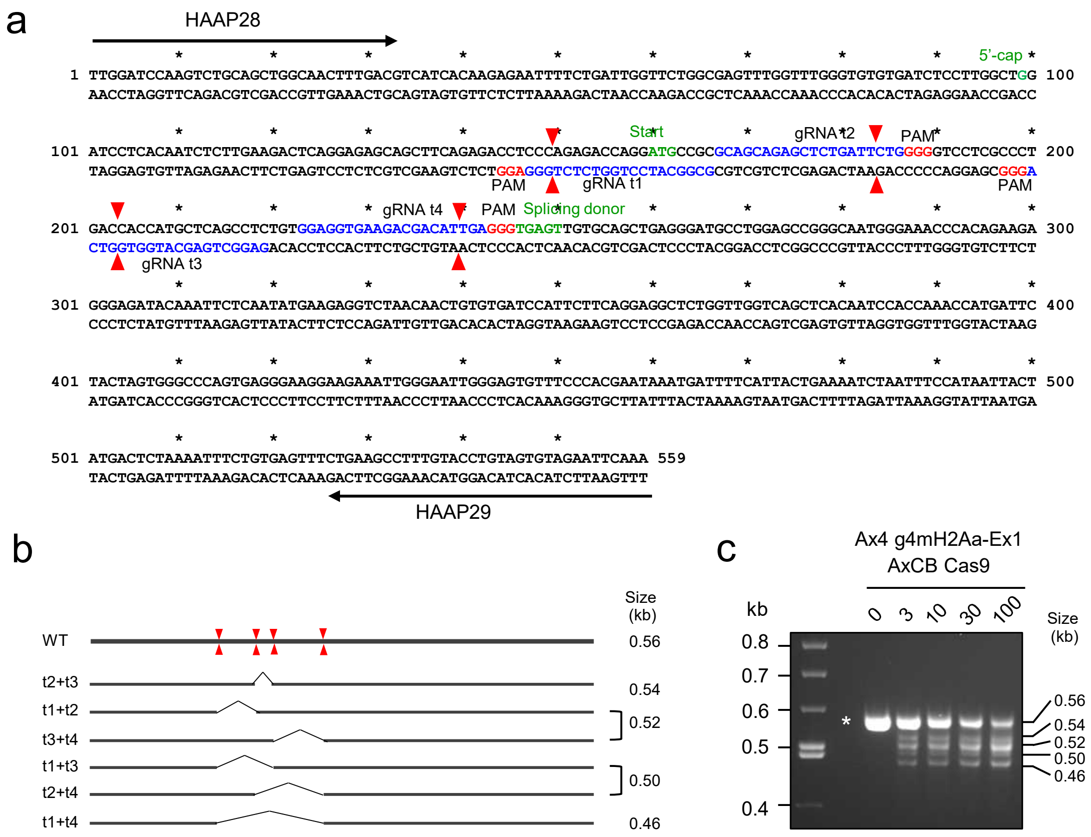

Supplementary Figure S4. Explanation for *H2-Aa* gene disruption using AdV expressing four multiplex gRNAs against *H2-Aa*. **(a)** Cleavage sites of the four gRNAs. The four gRNA target and PAM sequences of 5′-NGG are shown in blue and red, respectively. The cleavage positions (red triangle) are 3 nt upstream of the PAM sequences. The 5′-cap site, the start codon and the splicing donor site of exon 1 are shown in green. PCR primers (HAAP28 and HAAP29) used in C are shown as arrows. **(b)** Schematic representation of the possible gRNA cleavage sites (red triangle) in the PCR products. **(c)** Detailed explanation of the genome editing in MEF cells by infection of AdV (Ax4 g4mH2Aa-Ex1) expressing four multiplex gRNAs targeting *H2-Aa* (the same picture as in Fig. 2f). MEF cells were coinfected with the above AdV and the AdV expressing Cas9 (AxCB Cas9) at MOIs 3, 10, 30, and 100, and PCR products amplified using the cellular DNA by primers HAAP28 and HAAP29 are shown. The size of the four PCR products smaller than the intact fragment (0.56 kb) corresponded to the fragments derived from gene editing shown in b. Full-length gels are presented in Supplementary Figure S10.

Supplementary Figure S5

a

Site name	Sequences
H2-Aa t1	CCTCCCAGAGACCAGGATGCCGC
Off-target t1-1	CC**G**CCCAGAGACCAGGA**A**GC**T**GC
Off-target t1-2	CC**A**CCCAGAGACCAG**A**A**AT**CCGC
H2-Aa t3	CCCTGACCACCATGCTCAGCCTC
Off-target t3-1	CC**T**TGACCACCATGCTC**T**G**TG**TC
Off-target t3-2	CCCTGACCACCATGCT**G**AG**A**CT**T**

b

Off-target t1-1

5′-TCGTCGGCAGCGTCAGATGTGTATAAGAGACAGatcactgtgccctgtgaaagcagag-3′

5′-GTCTCGTGGGCTCGGAGATGTGTATAAGAGACAGaagaatgagtgacagaatcagaacc-3′

Off-target t1-2

5′-TCGTCGGCAGCGTCAGATGTGTATAAGAGACAGtcgtaaagtctcttaggtgtcagg-3′

5′-GTCTCGTGGGCTCGGAGATGTGTATAAGAGACAGggaaagttcaaaagggcatcaatcc-3′

Off target t3-1

5′-TCGTCGGCAGCGTCAGATGTGTATAAGAGACAGaagctaccctaaaagcaggctttgg-3′

5′-GTCTCGTGGGCTCGGAGATGTGTATAAGAGACAGtggcctgtcctgcgactcgactag-3′

Off-target t3-2

5′-TCGTCGGCAGCGTCAGATGTGTATAAGAGACAGgctagagaccttagtccgataac-3′

5′-GTCTCGTGGGCTCGGAGATGTGTATAAGAGACAGggaactggaactagagaaatgtg-3′

c

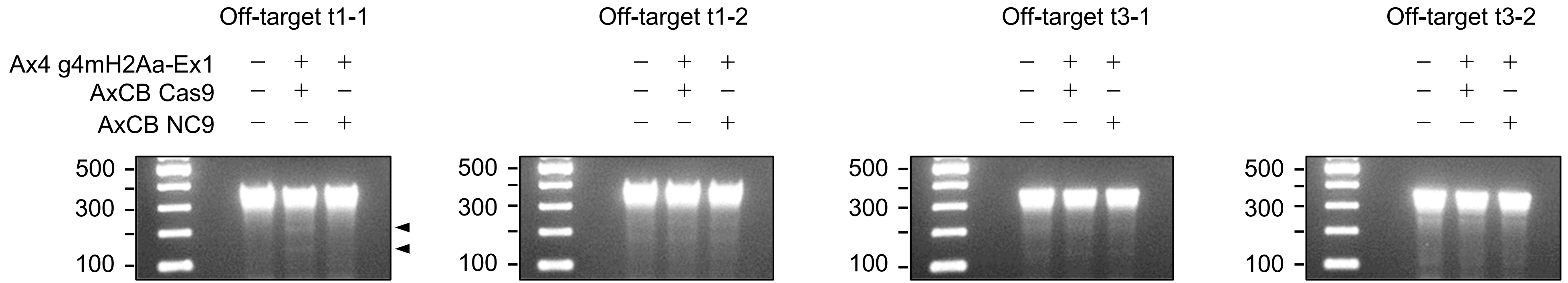

Supplementary Figure S5. Off-target analysis. **(a)** The target *H2-Aa* sequences shown in Fig. 3d and possible off-target sequences in the mouse genome. Underlining indicates the differences between the targeted *H2-Aa* sequences and the candidate off-target sequences. **(b)** The primers used for the detection of the sequences of the possible off-target sites. **(c)** Detection of off-target mutations. Total cellular DNAs isolated from mouse embryonic fibroblast cells infected with the AdVs expressing four multiplex gRNAs targeting *H2-Aa* (Ax4g4mH2Aa-Ex1) and Cas9/Cas9 nickase (AxCB Cas9/NC9) at MOI 0 and 100 (shown in Fig. 2f) were subjected to T7EI assays. Full-length gels are presented in Supplementary Figure S10.

Supplementary Figure S6

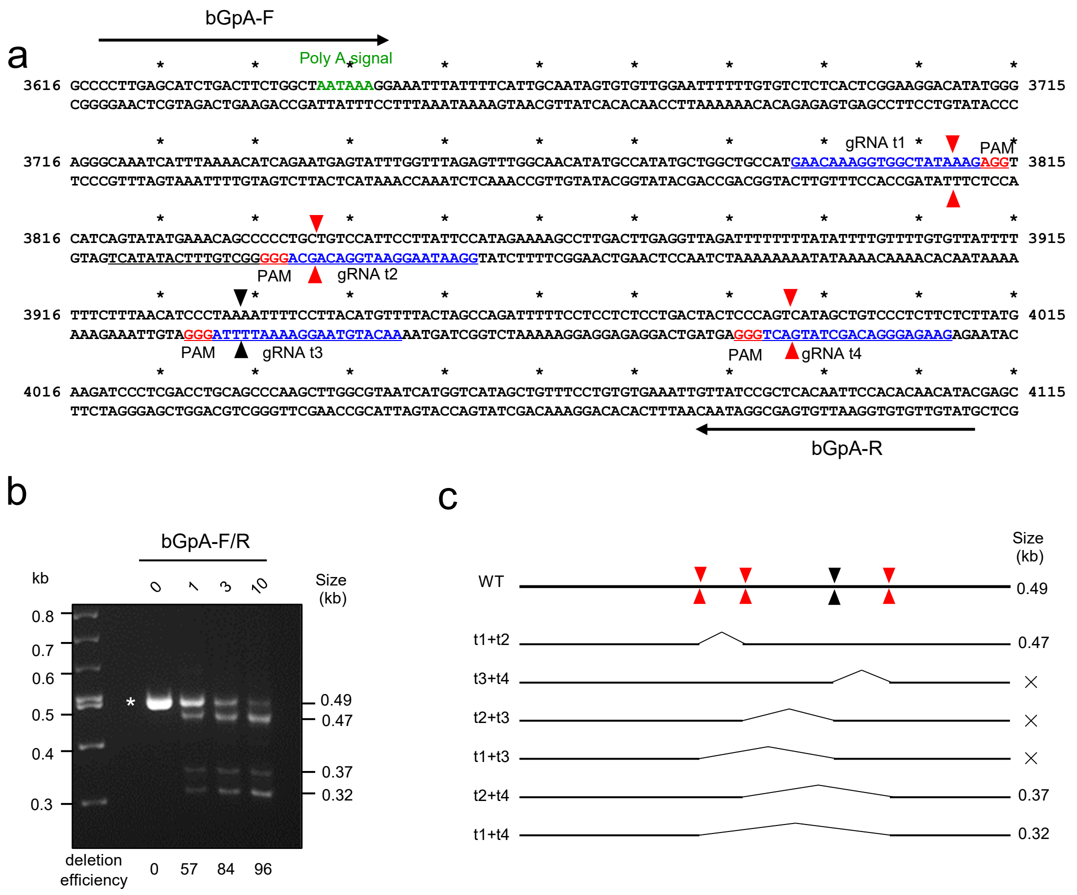

Supplementary Figure S6. Disruption of the chicken β-globin poly(A) region present in the chromosome of HepG2 cells using AdV expressing four multiplex gRNAs. (a) Representation of the poly(A) region targeted by the four multiplex gRNAs. The four gRNA targets and PAM sequences of 5′-NGG are shown in blue and red, respectively. The cleavage positions (red and black triangles) are 3 nt upstream of the PAM sequences. Note that the 5′ end of the gRNA target 3 (t3) was A instead of G, which would be the cause of low transcription of gRNA t3 (black triangle). PCR primers (bGpA-F and R) used in b are shown as arrows. **(b)** *In vitro* genome editing of the poly(A) region. The HepG2 cells were co-infected with the AdV expressing four multiplex gRNAs targeting poly(A) region and AdV expressing Cas9 at MOIs 1, 3, and 10, and were subjected to PCR using total cellular DNA 3 days after infection. An asterisk shows intact 0.49 kb PCR fragments of the target region. The size of the other three PCR products (0.47, 0.37, and 0.32 kb) smaller than that of the intact fragment corresponded to the fragments derived from gene editing shown in c. Deletion efficiency is shown as decrease in the percentage of the band intensity of 0.49-kb fragments compared to MOI 0. Full-length gels are presented in Supplementary Figure S10. **(c)** Schematic representation of possible gRNA cleavage sites (red and black triangle) in the PCR products. A black triangle indicates the inefficient cleavage site of gRNA t3 due to low gRNA transcription because no band produced by gRNA t3 (marked as ×) was observed.

Supplementary Figure S7

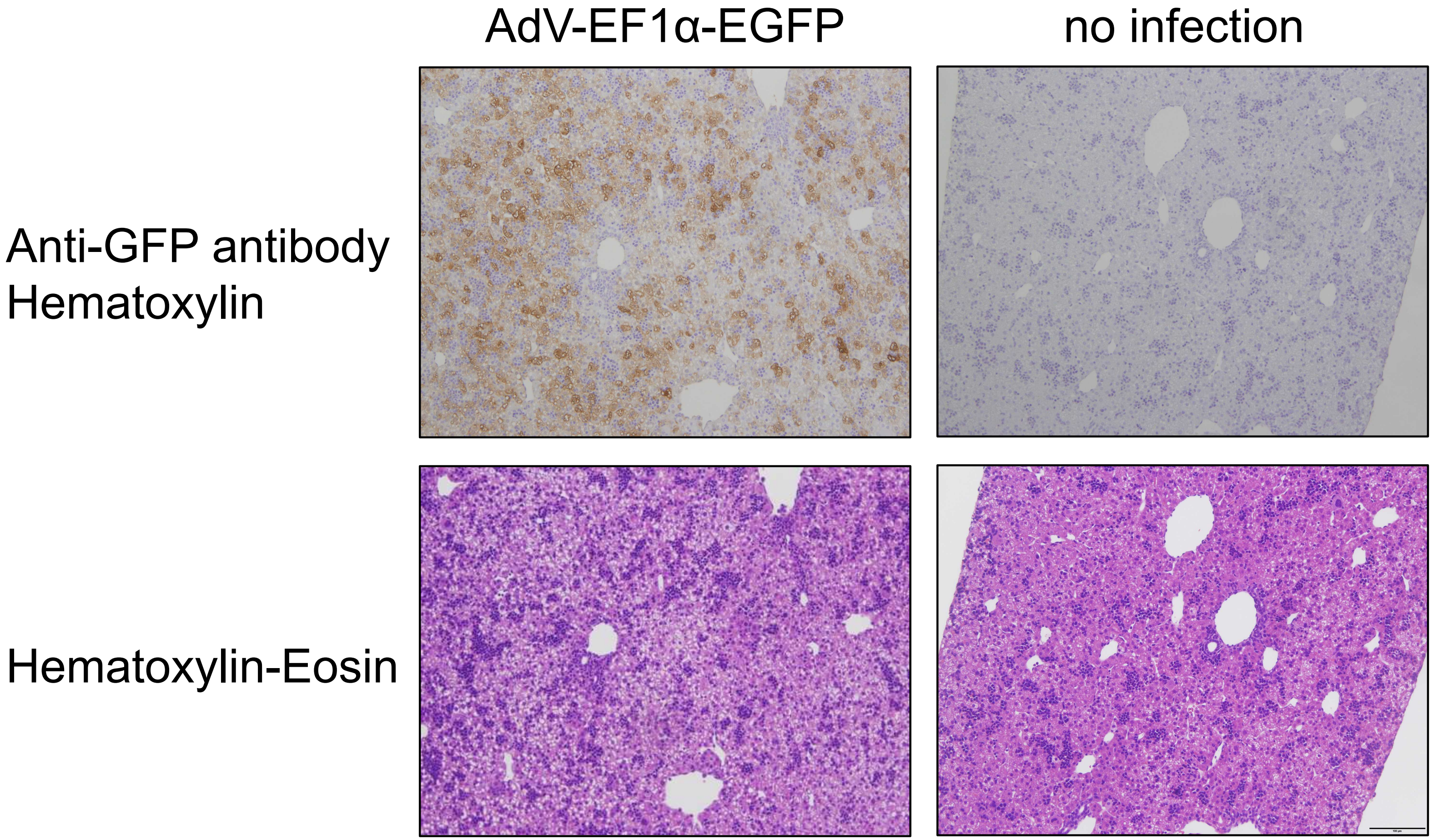

Supplementary Figure S7. Localization of EGFP expressing cells in liver from neonatal mice administered with AdV expressing EGFP under the control of EF1α promoter. A neonatal mouse administered at 0.5×10^8 $TCID_{50r}$ dose via the facial vein was dissected after 3 days and paraffin serial sections of liver were stained with an anti-GFP antibody followed by counter staining by Hematoxylin or with Hematoxylin-Eosin.

Supplementary Figure S8

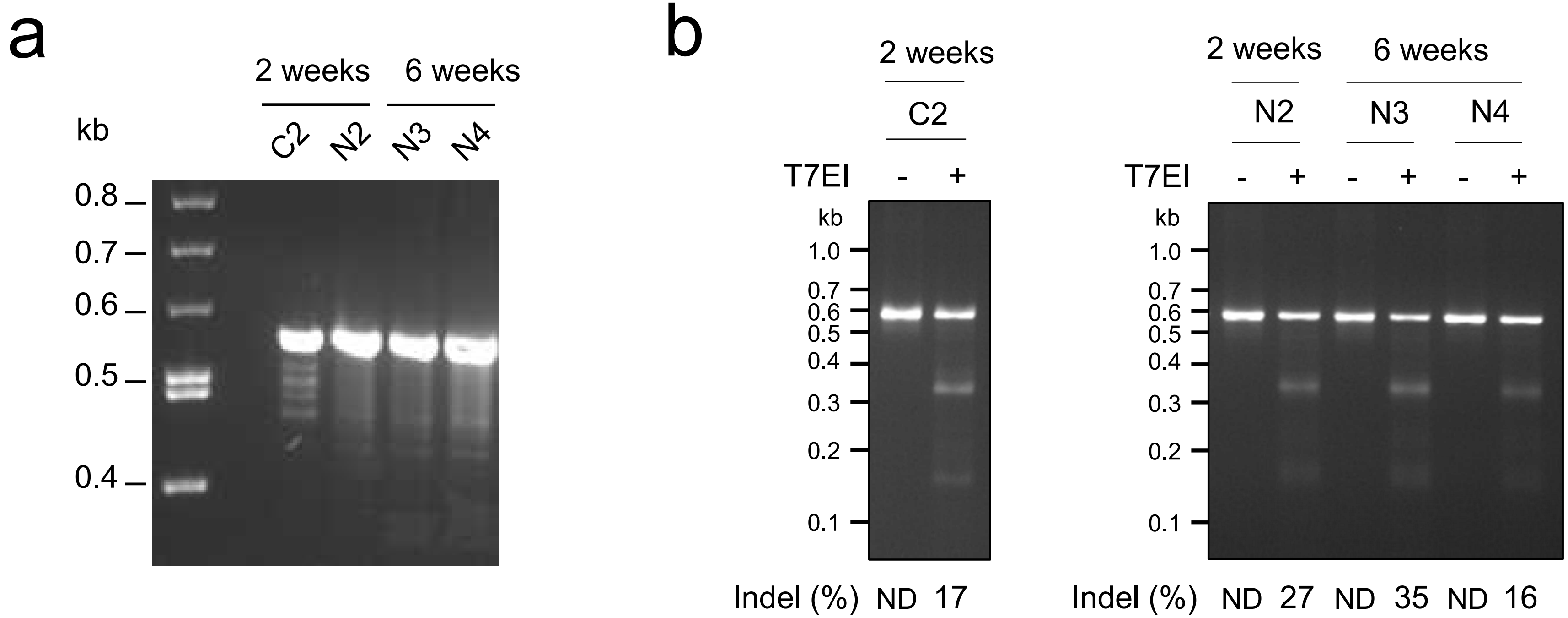

Supplementary Figure S8. Additional results of the genome editing in liver cells administered with AdV expressing four multiplex gRNAs targeting *H2-Aa*. **(a)** PCR analysis of the liver genomic DNA from neonatal mice administered by AdV expressing four multiplex gRNAs targeting *H2-Aa* (1.0×10^{8} $TCID_{50r}$ dose) together with AdV expressing Cas9 nickase or native Cas9 (1.0×10^{8} $TCID_{50r}$ dose). The liver genomic DNAs were isolated 2 (mouse C2 and N2) and 6 weeks (mouse N3 and N4) post-administration and were subjected to PCR analysis using primers HAAP 28 and 29 (Supplementary Fig. S4a). C2, mouse infected with AdVs expressing four multiplex gRNAs and Cas9: N2 to N4, mice infected with AdVs expressing four multiplex gRNAs and Cas9 nickase. **(b)** T7EI assays. The liver genomic DNAs isolated from mice C2 and N2 to N4 were subjected to T7EI assays. Indel frequencies are shown under the lanes. ND, not detected. Full-length gels are presented in Supplementary Figure S10.

Supplementary Figure S9

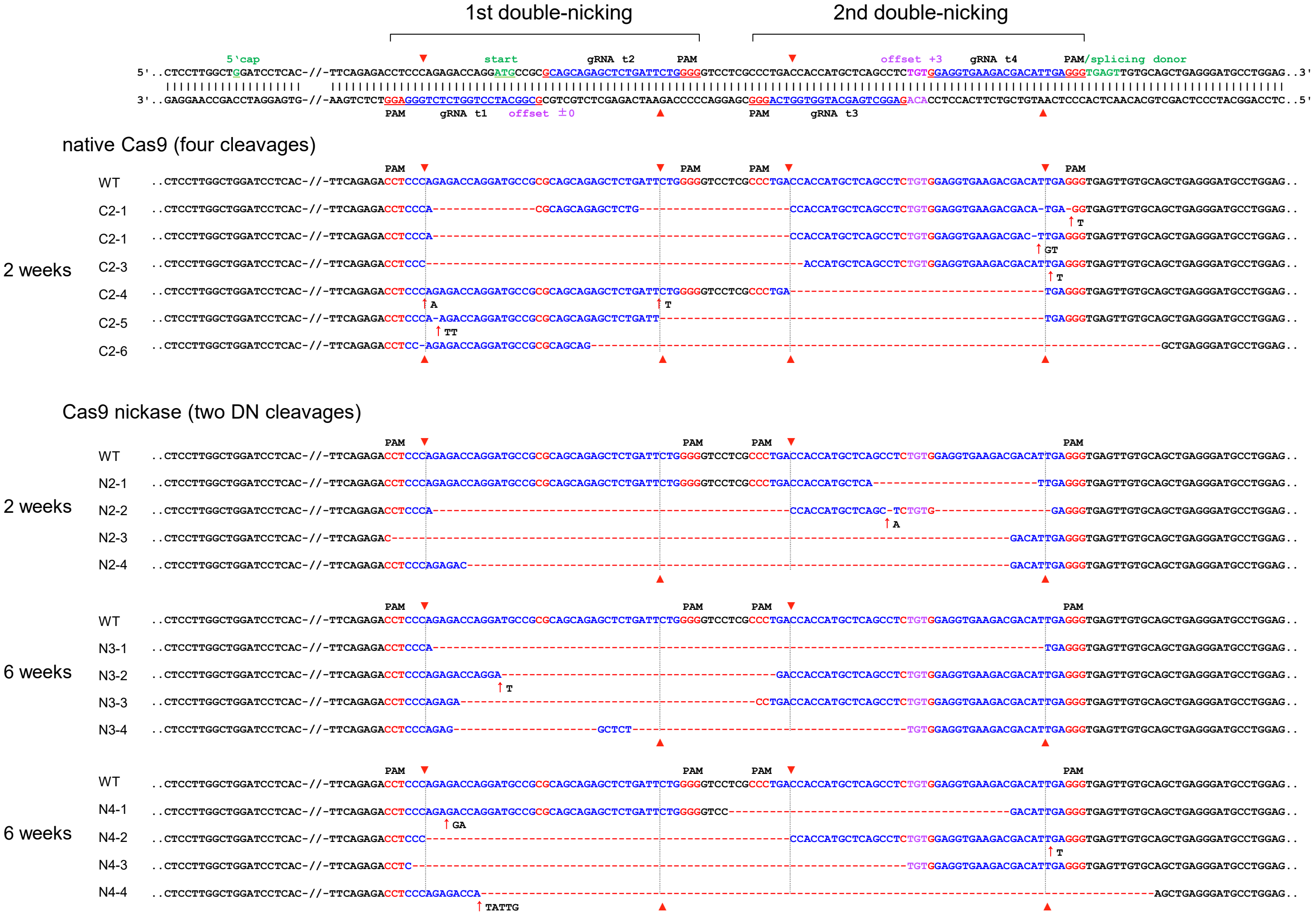

Supplementary Figure S9. Indel sequences in the two double-nicking target regions of the *H2-Aa* gene in additional mice. Liver cells were examined 2 or 6 weeks after administration of AdV expressing four multiplex gRNAs together with Cas9 or Cas9 nickase. (Upper row) The double-nicking target positions and sequences in exon 1 of the mouse *H2-Aa* gene. Representations are the same as in Fig. 3d. (Middle row) The indel sequences generated in liver cells from mouse administered with AdVs expressing four multiplex gRNAs and native Cas9. The mouse C2 was examined 2 weeks post administration. C2-1 to C2-6 are clones, which contained indels in 56 clones examined. (Lower row) The indel sequences generated in liver cells from mice administered with AdVs expressing four multiplex gRNAs and Cas9 nickase. Mouse N2 was examined 2 weeks after administration, and mouse N3 and N4 were examined 6 weeks after administration. N2-1 to N2-4, N3-1 to N3-4, and N4-1 to N4-4 are clones, which contained indels out of 56 clones examined from mouse N2, N3, and N4, respectively.

Supplementary Figure S10

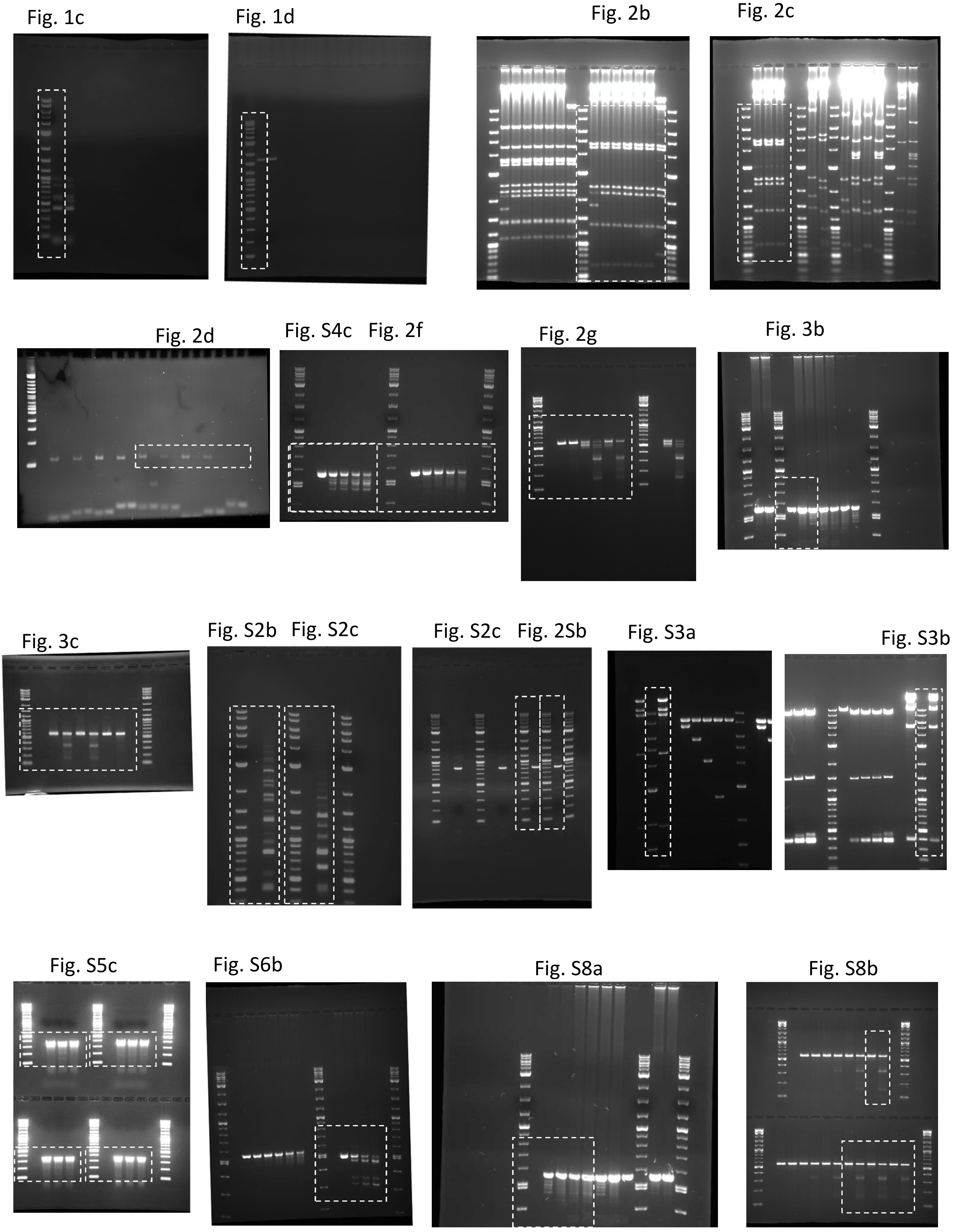

Supplementary Figure S10. Uncropped, unprocessed images
